# Maintenance of the functional integrity of mouse hematopoiesis by EED and promotion of leukemogenesis by EED haploinsufficiency

**DOI:** 10.1038/srep29454

**Published:** 2016-07-19

**Authors:** Kenichiro Ikeda, Takeshi Ueda, Norimasa Yamasaki, Yuichiro Nakata, Yasuyuki Sera, Akiko Nagamachi, Takahiko Miyama, Hiroshi Kobayashi, Keiyo Takubo, Akinori Kanai, Hideaki Oda, Linda Wolff, Zen-ichiro Honda, Tatsuo Ichinohe, Akio Matsubara, Toshio Suda, Toshiya Inaba, Hiroaki Honda

**Affiliations:** 1 Department of Disease Model, Research Institute for Radiation Biology and Medicine, Hiroshima University, 1-2-3 Kasumi, Minami-ku, Hiroshima 734-8553, Japan; 2 Department of Urology, Institute of Biomedical and Health Sciences, Hiroshima University, 1-2-3 Kasumi, Minami-ku, Hiroshima 734-8553, Japan; 3 Department of Molecular Oncology, Research Institute for Radiation Biology and Medicine, Hiroshima University, 1-2-3 Kasumi, Minami-ku, Hiroshima 734-8553, Japan; 4 Department of Hematology and Oncology, Research Institute for Radiation Biology and Medicine, Hiroshima University, 1-2-3 Kasumi, Minami-ku, Hiroshima 734-8553, Japan; 5 Department of Stem Cell Biology, Research Institute, National Center for Global Health and Medicine, 1-21-1, Toyama, Shinjuku-ku, Tokyo 162-8655, Japan; 6 Department of Pathology, Tokyo Women’s Medical University, 8-1 Kawada-cho, Shinjuku-ku, Tokyo 162-8666, Japan; 7 Laboratory of Cellular Oncology, Center for Cancer Research, National Cancer Institute, Bethesda, MD, USA; 8 Health Care Center and Graduate School of Humanities and Sciences, Institute of Environmental Science for Human Life, Ochanomizu University, 2-1-1 Otsuka Bunkyo-ku, Tokyo 112-8611, Japan; 9 Cancer Science Institute of Singapore, National University of Singapore Center for Translational Medicine, 14 Medical Drive, #12-01, 117599, Singapore

## Abstract

Polycomb repressive complex 2 (PRC2) participates in transcriptional repression through methylation of histone H3K27. The WD-repeat protein embryonic ectoderm development (EED) is a non-catalytic but an essential component of PRC2 and its mutations were identified in hematopoietic malignancies. To clarify the role(s) of EED in adult hematopoiesis and leukemogenesis, we generated *Eed* conditional knockout (*Eed*^*Δ*/*Δ*^) mice. *Eed*^*Δ*/*Δ*^ mice died in a short period with rapid decrease of hematopoietic cells. Hematopoietic stem/progenitor cells (HSPCs) were markedly decreased with impaired bone marrow (BM) repopulation ability. Cell cycle analysis of HSPCs demonstrated increased S-phase fraction coupled with suppressed G0/G1 entry. Genes encoding cell adhesion molecules are significantly enriched in *Eed*^*Δ*/*Δ*^ HSPCs, and consistently, *Eed*^*Δ*/*Δ*^ HSPCs exhibited increased attachment to a major extracellular matrix component, fibronectin. Thus, EED deficiency increases proliferation on one side but promotes quiescence possibly by enhanced adhesion to the hematopoietic niche on the other, and these conflicting events would lead to abnormal differentiation and functional defect of *Eed*^*Δ*/*Δ*^ HSPCs. In addition, *Eed* haploinsufficiency induced hematopoietic dysplasia, and *Eed* heterozygous mice were susceptible to malignant transformation and developed leukemia in cooperation with *Evi1* overexpression. Our results demonstrated differentiation stage-specific and dose-dependent roles of EED in normal hematopoiesis and leukemogenesis.

Epigenetic mechanisms of gene regulation are required for proper stem cell function and differentiation, and its deregulation contributes to malignant transformation^1^,^2^,^3^. Tri-methylation of Lys 4 and Lys 27 residues in histone H3 (H3K4me3 and H3K27me3) is considered to be the activation and silencing histone modification, respectively^4^. In embryonic stem cells, these histone modifications coexist at developmental regulator gene promoters (so-called bivalent domains) to maintain the genes in an activation ready state^5^. In response to various stimuli, promoters with bivalent domains are resolved into a monovalent state, either H3K4me3 or H3K27me3, which activates or suppresses gene expression profiles and leads to cell differentiation. Recent findings have revealed that bivalent domains also exist in hematopoietic stem cells (HSCs); therefore, these two histone modifications are considered to be crucial for proper maintenance and functional integrity of HSCs^6^.

Polycomb repressive complex 2 (PRC2) catalyzes H3K27me3, a repressive histone marker of gene silencing^7^. PRC2 comprises 3 core subunits: EZH2, EED, and SUZ12; EZH2 functions as a methyltransferase, whereas the other subunits are non-catalytic. EED directly interacts with EZH2 and enhances its methyltransferase activity^8^. In addition, EED binds to H3K27me3 through its aromatic cage residues, thereby promoting the allosteric activation of PRC2 and propagating H3K27me3^9^. Through these functions, EED plays an essential role in the full exertion of the catalytic activity of PRC2.

Clinically, loss-of-function mutations of these PRC2 components have been identified in human hematopoietic malignancies of the myeloid and T-cell lineages^10^,^11^. We previously reported *Eed* gene mutations resulting in impaired PRC2 function (deletions and/or point mutations) in myelodysplastic syndrome (MDS) and related diseases^12^. We demonstrated that all mutated forms of EED exhibited functional defects involving protein stability, impaired interactions with EZH2, and/or binding to H3K27me3^12^. Therefore, dysregulated PRC2 functions, including EED, has been proposed to be associated with the pathogenesis of hematopoietic malignancies.

In this study, we generated and analyzed tamoxifen-inducible conditional *Eed* knockout mice to investigate the role of EED in normal hematopoiesis and leukemogenesis.

## Results

### Acquired deletion of EED results in PRC2 dysfunction and induced premature death associated with hematopoietic failure

**Figure 1:**
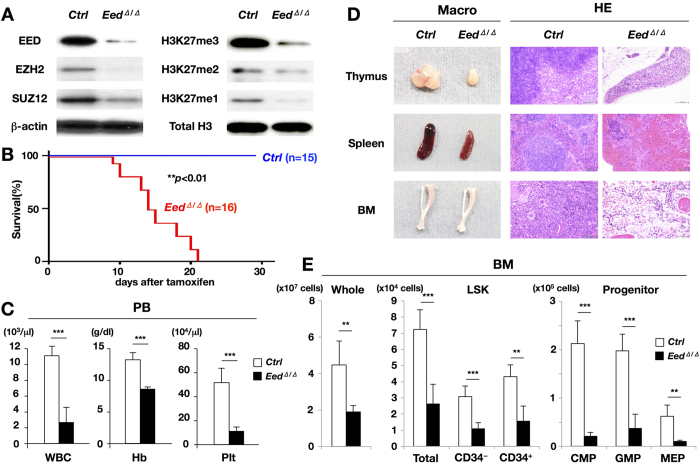
Analysis of *Eed*^*Δ*/*Δ*^ mice. (**A**) Western blot of EED and other PRC2 components, EZH2, and SUZ12 (left panels), and H3K27me3–me1 (right panels) in the spleens of *control* and *Eed*^*Δ*/*Δ*^ mice. Note that the expression of not only EED but also EZH2 and SUZ12 was decreased in *Eed*^*Δ*/*Δ*^ spleens, accompanied by a marked reduction in H3K27me3–me1. (**B**) Survival curves of *control* and *Eed*^*Δ*/*Δ*^ mice. *Eed*^*Δ*/*Δ*^ mice died within 3 weeks after tamoxifen administration. (**C**) PB parameters in *control* and *Eed*^*Δ*/*Δ*^ mice at 2 weeks after tamoxifen administration. *Eed*^*Δ*/*Δ*^ mice exhibited a marked decrease in white blood cell (WBC) counts, hemoglobin (Hb) concentrations, and platelet (Plt) numbers relative to *control* mice. ***p < 0.001. (**D**) Macroscopic appearances and hematoxylin and eosin-stained sections of hematopoietic tissues (thymus, spleen, and BM) from *control* and *Eed*^*Δ*/*Δ*^ mice. Note the marked hypoplasia and decrease in hematopoietic cells in *Eed*^*Δ*/*Δ*^ mice. (**E**) Cell numbers of whole, LSK (total, CD34^−^, and CD34^+^), and progenitor (CMP, GMP, and MEP) fractions in the BM of *control* and *Eed*^*Δ*/*Δ*^ mice at 2 weeks after tamoxifen administration. Cell numbers in all the fractions were significantly reduced in *Eed*^*Δ*/*Δ*^ mice. **p < 0.01, ***p < 0.001.

To conditionally ablate EED function, we generated mice in which exon 6 of the *Eed* gene was *floxed* (Supplementary Fig. 1A). Correctly targeted ES cells identified via Southern blotting with 5′ and 3′ genomic probes (Supplementary Fig. 1B) were used to create chimeric mice that transmitted the mutated allele through the germline. Mice carrying the *floxed* allele (*Eed*^*flox*/*flox*^) were crossed with *Cre*^*ERT2*+^ mice, in which Cre is inducibly activated by tamoxifen. Western blot (WB) analysis demonstrated that the expression of EED protein was markedly decreased in the spleen of a tamoxifen-treated *Eed*^*flox*/*flox*^, *Cre*^*ERT2*+^ mouse compared with that in a tamoxifen-treated *Eed*^*flox*/*flox*^, *Cre*^*ERT2*−^ mouse (Fig. 1A, left top panel). To more accurately assess the deletion level of EED in hematopoietic cells, anti-EED WB was performed using bone marrow (BM) cells. As shown in Supplementary Fig. 2A, EED protein was almost undetectable in tamoxifen-treated *Eed*^*flox*/*flox*^, *Cre*^*ERT2*+^ BM cells. In addition, RNA sequencing revealed that *Eed* exon 6-derived transcript was almost completely absent in tamoxifen-treated *Eed*^*flox*/*flox*^, *Cre*^*ERT2*+^ hematopoietic cells (indicated by an arrow in Supplementary Fig. 2B). These findings indicated that our targeting strategy successfully ablated the *Eed* gene product (thus, hereafter tamoxifen-treated *Eed*^*flox*/*flox*^, *Cre*^*ERT2*−^ and *Eed*^*flox*/*flox*^, *Cre*^*ERT2*+^ mice are referred to as *control* and *Eed*^*Δ*/*Δ*^ mice, respectively). The expression levels of other PRC2 components, EZH2 and SUZ12, were also considerably decreased in the *Eed*^*Δ*/*Δ*^ spleen (Fig. 1A, left 2^nd^ and 3^rd^ panels). In accordance with these observations, the tri-methylation level of H3K27 (H3K27me3) was markedly reduced, along with decreases in the di- and mono-methylation levels of H3K27 (H3K27me2 and H3K27me1; Fig. 1A, right panels).

*Eed*^*Δ*/*Δ*^ mice rapidly became emaciated and died within 3 weeks of tamoxifen administration (Fig. 1B). Examination of peripheral blood (PB) parameters revealed a significant reduction in all hematopoietic lineages, including white blood cell (WBC) counts, hemoglobin (Hb) concentrations, and platelet (Plt) numbers, in *Eed*^*Δ*/*Δ*^ mice (Fig. 1C). Macroscopic and pathological analysis of *Eed*^*Δ*/*Δ*^ mice revealed marked thymic and splenic atrophy and pale appearance of the BM, in which the number of hematopoietic cells was significantly reduced (Fig. 1D). Despite a detailed pathological examination, no obvious changes to which death could be attributed were detected in other major organs, strongly suggesting that *Eed*^*Δ*/*Δ*^ mice died of hematopoietic failure. The analysis of hematopoietic stem-progenitor cells (HSPCs) in the BM revealed that cell numbers of whole, primitive HSC-containing LSK (Lin^−^, Sca-1^+^, c-kit^+^) fractions (Total, CD34^−^, and CD34^+^), and more differentiated progenitor fractions (CMP, GMP, and MEP) were significantly reduced in *Eed*^*Δ*/*Δ*^ mice relative to *controls* (Fig. 1E), with the reduction levels being a little milder in LSK fractions than in progenitor fractions. These results indicate that EED plays an essential role in maintaining the functional integrity of adult hematopoiesis by stabilizing PRC2 and maintaining the global methylation status of H3K27.

### Impaired reconstitution ability of *Eed* ^*Δ*/*Δ*^ HSPCs

**Figure 2:**
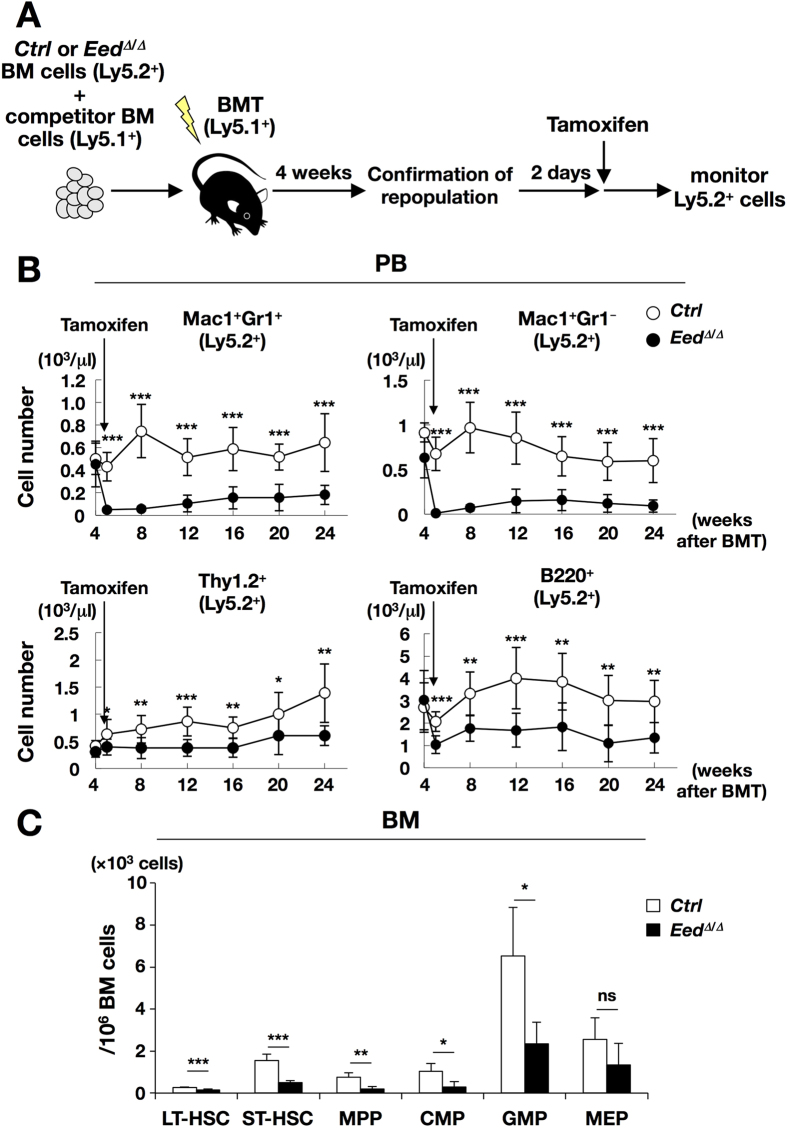
Competitive repopulation assay in *Eed*^*Δ*/*Δ*^ mice. (**A**) Experimental procedure of the competitive repopulation assay. (**B**) Chimerism of lineage-committed donor cells in the PB. *p < 0.05, **p < 0.01, ***p < 0.001. (**C**) Analysis of donor-derived ratios of HSPCs in the bone marrow (BM). ns; not significant, *p < 0.05, **p < 0.01, ***p < 0.001.

To further investigate the effect of EED deficiency on HSPC function *in vivo*, a competitive repopulation assay using BM transplantation (BMT) was performed. As *Eed*^*Δ*/*Δ*^ mice rapidly became emaciated following tamoxifen administration, we first transplanted Ly5.2^+^ donor (*Eed*^*flox*/*flox*^, *Cre*^*ERT2*−^ and *Eed*^*flox*/*flox*^, *Cre*^*ERT2*+^ mice) cells with competitor (Ly5.1^+^) cells into recipient (Ly5.1^+^) mice before tamoxifen administration to avoid defect of engraftment. Then, we confirmed repopulation of the transplanted cells at 4 weeks after BMT, and administered tamoxifen 2 days later (Fig. 2A). PB analysis revealed that chimerism of *Eed*^*Δ*/*Δ*^ cells was significantly lower than that of *control* cells in all lineages (Fig. 2B). In addition, BM examination revealed a significant reduction in almost all HSPC and myeloid fractions, including LT-HSC, ST-HSC, MPP, CMP, and GMP (Fig. 2C). These results indicated that repopulation ability of *Eed*^*Δ*/*Δ*^ HSPCs was significantly impaired, and the decrease in hematopoietic cells in *Eed*^*Δ*/*Δ*^ mice was mainly due to a cell-intrinsic mechanism.

### Increased cell proliferation and reduced cell cycle entry of *Eed* ^*Δ*/*Δ*^ HSPCs

To clarify the early primary event(s) in HSPCs induced by EED deficiency, *control* and *Eed*^*Δ*/*Δ*^ mice were analyzed 2 days after tamoxifen administration. The number of whole BM cells was significantly lower in *Eed*^*Δ*/*Δ*^ mice than in *control* mice (Supplementary Fig. 3, left panel), indicating an already evident decrease in hematopoietic cells at this stage. However, unexpectedly, the cell numbers of primitive LSK fractions in *Eed*^*Δ*/*Δ*^ mice, especially in the CD34^+^ fraction, was significantly higher than those in *controls* (Supplementary Fig. 3A, middle panel), while those of more differentiated CMP and MEP fractions in *Eed*^*Δ*/*Δ*^ mice were significantly decreased compared with those in *controls* (Supplementary Fig. 3A, right panel).

**Figure 3:**
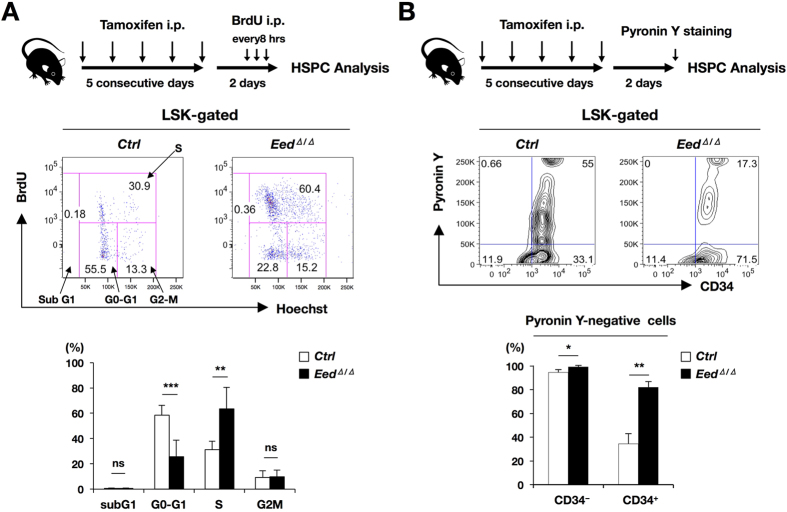
Cell cycle assays of *Eed*^*Δ*/*Δ*^ mice. (**A**) BrdU incorporation analysis. The experimental procedure, representative results from *control* and *Eed*^*Δ*/*Δ*^ LSK cells, and percentages of each cell-cycle fractions are shown. ns; not significant, **p < 0.01, ***p < 0.001. (**B**) Pyronin Y fluorescence measurement assay. The experimental procedure, representative results from *control* and *Eed*^*Δ*/*Δ*^ LSK cells, and percentages of Pyronin Y-negative cells in CD34^−^ and CD34^+^ fractions are shown. *p < 0.05, **p < 0.01.

We next analyzed the cell cycle status of HSPCs from *control* and *Eed*^*Δ*/*Δ*^ mice using flow cytometry. A BrdU (5-bromodeoxyuridine) incorporation analysis showed significant increase in the S phase in *Eed*^*Δ*/*Δ*^ LSK cells and a resulting significant decrease in the G0-G1 phase relative to *control* cells (Fig. 3A). These results indicate that EED deficiency increased proliferation at an early stage after tamoxifen. We then performed Pyronin Y staining to directly elucidate the G0–G1 transition in CD34^−^ and CD34^+^ LSK cells. In the CD34^−^ fraction, a difference in the Pyronin Y-negative ratio was not observed between *control* and *Eed*^*Δ*/*Δ*^ cells; on the other hand, in the CD34^+^ fraction, *Eed*^*Δ*/*Δ*^ cells exhibited a significantly higher Pyronin Y-negative ratio relative to *control* cells (Fig. 3B). These findings indicate that EED deficiency impaired normal cell cycle progression by promoting proliferative activity on one hand and inhibiting cell cycle entry on the other, leading to a compromise in the balance between HSPC quiescence and proliferation.

### *Eed* ^*Δ*/*Δ*^ HSPCs exhibited enhanced adherence to fibronectin coupled with increased expression of cell adhesion-associated genes

**Figure 4:**
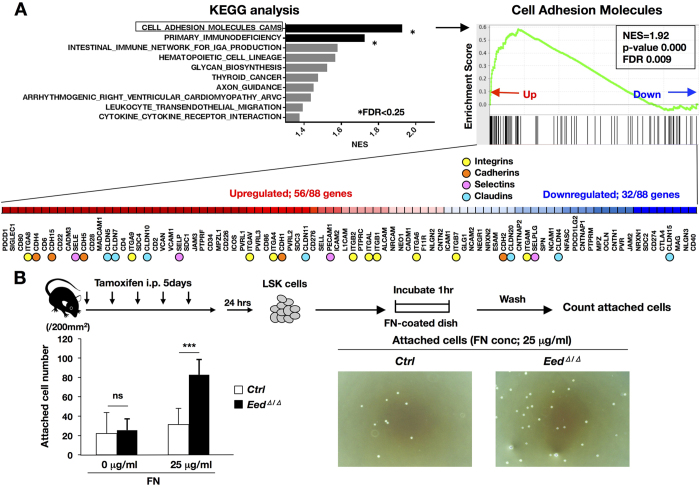
Transcriptome analysis and cell adhesion assay of *Eed*^*Δ*/*Δ*^ cells. (**A**) Results of a transcriptome analysis. The Top10 KEGG pathways enriched in *Eed*^*Δ*/*Δ*^ LSK cells, GSEA enrichment plot in the cell adhesion molecules gene set, and expressional changes in constituting genes are shown. Genes belonging to the integrin, cadherin, selectin and claudin families are indicated by circles. (**B**) Results of a cell adhesion assay. The experimental procedure, numbers of attached cells on dishes coated with different concentrations of fibronectin (FN; 0 and 25 μg/ml), and representative photos of attached cells at 25 μg/ml FN are shown. ns; not significant, ***p < 0.001.

To elucidate the molecular mechanisms responsible for the abnormal behavior of *Eed*^*Δ*/*Δ*^ HSPCs, LSK cells extracted from *control* and *Eed*^*Δ*/*Δ*^ mice 2 days after tamoxifen administration were subjected to RNA sequencing. As EED is a component of PRC2 that negatively regulates gene transcription, we expected that most differentially expressed genes in *Eed*^*Δ*/*Δ*^ LSK cells would be upregulated. However, the similar numbers of upregulated and downregulated genes were observed in early response to EED deficiency (Supplementary Fig. 4). To search for differentially regulated functional networks in *Eed*^*Δ*/*Δ*^ HSPCs, GSEA was applied to Kyoto Encyclopedia of Genes and Genomes (KEGG) pathway. As a result, we identified genes involved in Cell Adhesion Molecules, including integrins, cadherins, selectins, and claudins, as the top upregulated pathway (NES = 1.92, FD < 0.01) in *Eed*^*Δ*/*Δ*^ LSK (Fig. 4A). Enrichment in cell adhesion-associated genes was previously reported to define HSCs as a quiescence state^13^. Fibronectin interacts with integrins as an ECM component, and it is also a major constituent of hematopoietic niche^14^. We therefore investigated the adhesion ability of HSPCs to fibronectin using *in vitro* cell adhesion assay^15^. As shown in Fig. 4B, at 25 μg/ml of fibronectin, *Eed*^*Δ*/*Δ*^ LSK cells exhibited a significantly higher adhesion ability than *control* cells while there was no difference between two groups without fibronectin. Thus, *Eed* deficiency potentially promotes HSPCs to cling to the hematopoietic niche.

### *Eed* haploinsufficiency induced hematopoietic dysplasia resembling to MDS

Previous studies, including ours, reported somatic and hemiallelic mutations of *Eed* in MDS and related myeloid neoplasms^12^,^16^,^17^, strongly suggesting that reduced *Eed* expression might also affect hematopoiesis. We attempted to address this possibility using *Eed* heterozygous (*Eed*^+/*Δ*^) mice and *EED siRNA*-transduced human HSPCs.

**Figure 5:**
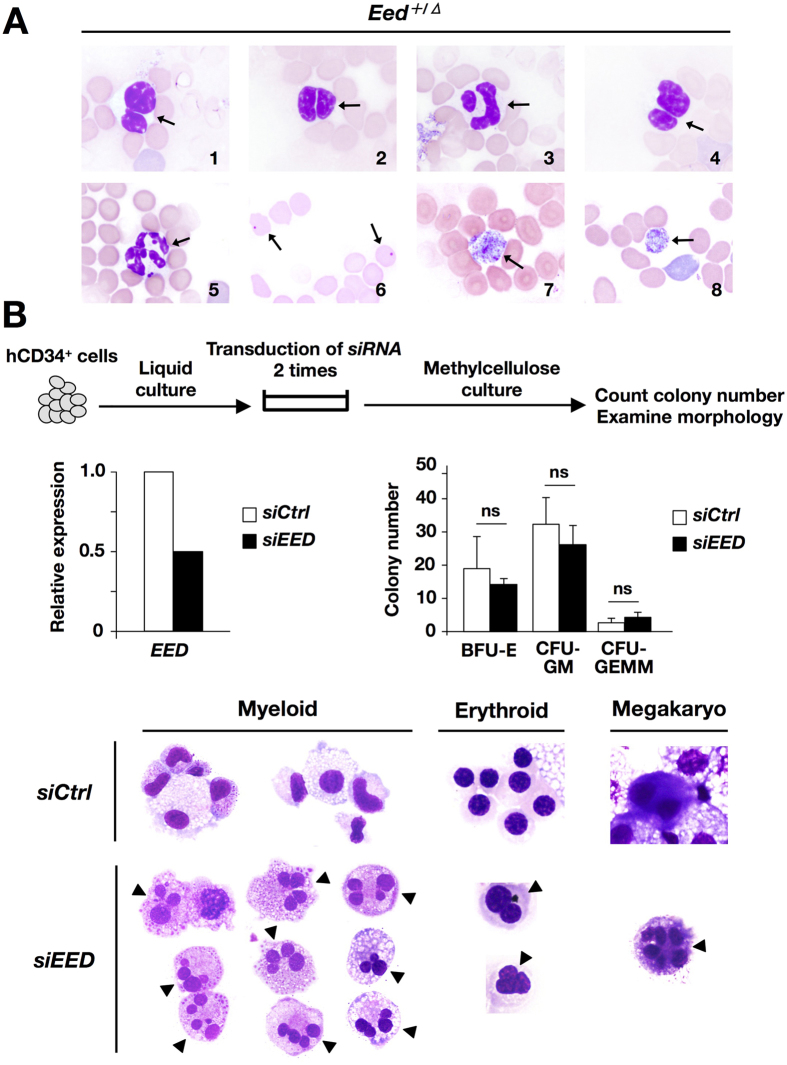
Morphological abnormalities in *Eed*^+/*Δ*^ PB cells and *siEED*-transduced human CD34^+^ cord blood cells. (**A**) Dysplastic cells in the PB smears of *Eed*^+/*Δ*^ mice at 1 year of age. Panels 1 to 4; WBCs with Pseudo-Pelger-Huet anomaly and abnormal nuclei (indicated by arrows), panel 5; a hypersegmented neutrophil (indicated by an arrow), panels 6; erythrocytes with a Howell-Jolly body (indicated by arrows), and panels 7 and 8; giant platelets (indicated by arrows). (**B**) Experimental procedure for transduction of *siRNA* in human CD34^+^ cord blood cells, and result of qPCR for *EED* expression, colony numbers, and Giemsa staining of *siCtrl*- and *siEED*-transduced cells. Morphological abnormalities were detected in the myeloid, erythroid, and megakaryocytic lineages in *siEED*-transduced cells (indicated by arrowheads). BFU-E; erythroid burst-forming units, CFU-GM; granulocyte-macrophage colony-forming units, CFU-GEMM; granulocyte/erythrocyte/macrophage/megakaryocyte colony-forming units.

In *Eed*^+/*Δ*^ mice, the EED and EZH2 expression levels were reduced by approximately half whereas those of SUZ12 and H3K27 were slightly decreased in *Eed*^+/*Δ*^ mice compared with *controls* (Supplementary Fig. 5A). Although no apparent changes were observed in the cell number in HSPC fractions (Supplementary Fig. 5B), PB smears of *Eed*^+/*Δ*^ mice at 1 year of age occasionally exhibited hematopoietic dysplasia, such as WBCs with abnormal nuclei including Pseudo-Pelger-Huet anomaly, hypersegmented neutrophils, erythrocytes with a Howell-Jolly body, and giant platelets (Fig. 5A), which were not observed in *control* littermates.

We also investigated morphological changes induced by suppressive expression of *EED* using human HSPCs. To this end, human CD34^+^ cord blood cells were transduced with *control* or *EED siRNA*, cultured in methylcellulose containing cytokines, and subjected to colony counting and morphological examination (Fig. 5B, upper panel). qPCR analysis revealed that the expression level of *EED* was reduced by approximately half in *EED siRNA*-transduced CD34^+^ (*siEED*) cells compared with *control siRNA*-transduced CD34^+^ (*siCtrl*) cells (Fig. 5B, left middle panel), thus mimicking *Eed* haploinsufficiency. Although no apparent difference was observed in the colony numbers between both types of cells at 14 days after plating (Fig. 5B, right middle panel), *siEED* cells exhibited various morphological abnormalities such as multiple, dispersed, and asymmetrically divided nuclei in the myeloid, erythroid, and megakaryocytic lineages, which were not detected in *siCtrl* cells (Fig. 5B, lower panels). These findings demonstrated that haploinsufficiency of *Eed*/*EED* in HSPCs induced hematopoietic dysplasia resembling to MDS.

### *Eed* haploinsufficiency conferred a proliferative advantage and rendered enhanced susceptibility to leukemic transformation

We finally investigated the contribution of *Eed* haploinsufficiency to leukemia predisposition. We first examined the proliferative ability of *Eed*^+/*Δ*^ hematopoietic cells by a competitive repopulation assay. As shown in Supplementary Fig. 6, *Eed*^+/*Δ*^ HSPCs exhibited higher chimerism than *control* cells on average, with a significant increase in the PB at the early phase (1–3 months after BMT).

**Figure 6:**
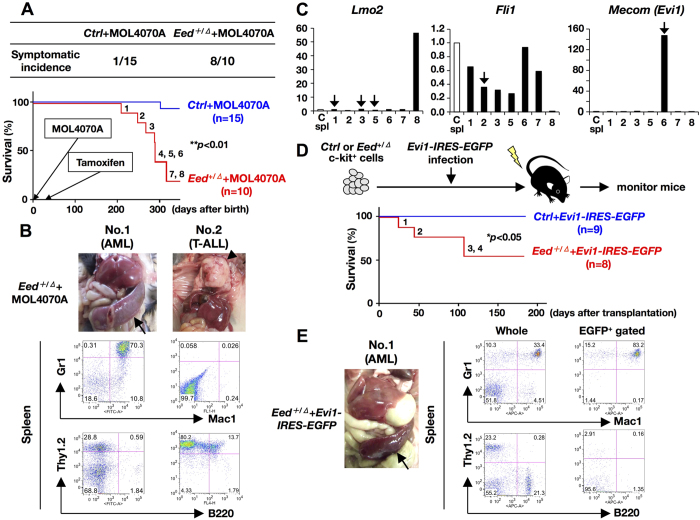
Leukemia susceptibility of *Eed*^+/*Δ*^ mice. (**A**) Symptomatic incidence and survival curves of *Ctrl* + MOL4070A and *Eed*^+/*Δ*^ + MOL4070A mice. *Eed*^+/*Δ*^ + MOL4070A mice exhibited significantly higher morbidity and mortality compared with *Ctrl* + MOL4070A mice (**p < 0.01). Diseased mice in the *Eed*^+/*Δ*^ + MOL4070A group are numbered. (**B**) Representative results of macroscopic appearances and FACS analysis of *Eed*^+/*Δ*^ + MOL4070A leukemic mice with AML (No. 1, left panel) and T-ALL (No. 2, right panel). Splenomegaly and thymoma observed in No. 1 and No. 2 are indicated by an arrow and an arrowhead, respectively. (**C**) Expression analysis of *Lmo2, Fli1*, and *MECOM (Evi1*) in a control spleen (C Spl) and *Eed*^+/*Δ*^ + MOL4070A leukemic spleens (No. 1–3, and 5–8). Arrows indicate tumor sample(s) in which retroviral integration was detected by iPCR. (**D**) Cooperative ability of *Eed* haploinsufficiency and *Evi1* overexpression to induce AML. The experimental procedure and survival curves of *Evi1-IRES-EGFP*-transduced *control* and *Eed*^+/*Δ*^ mice (*Ctrl* + *Evi1-IRES-EGFP* and *Eed*^+/*Δ*^ + *Evi1-IRES-EGFP*) are shown. Diseased mice in the *Eed*^+/*Δ*^ + *Evi1-IRES-EGFP* group are numbered. (**E**) Representative results of macroscopic and FACS analyses of an *Eed*^+/*Δ*^ + *Evi1-IRES-EGFP* leukemic mouse with AML are shown. Enlarged spleen is indicated by an arrow.

To further analyze the susceptibility of *Eed*^+/*Δ*^ mice to leukemia, we applied retrovirus-mediated insertional mutagenesis by using MOL4070LTR retrovirus (MOL4070A), which integrates into the mouse genome and upregulates the expression of neighboring genes^18^. During a 1-year observation period, 8 of 10 MOL4070A-infected *Eed* heterozygotes (*Eed*^+/*Δ*^ + MOL4070A) developed acute leukemia, whereas only 1 of 15 MOL4070A-infected *controls (Ctrl* + MOL4070A) exhibited hematopoietic abnormalities (Fig. 6A, *p* < 0.01). Macroscopically, all diseased *Eed*^+/*Δ*^ + MOL4070A mice exhibited massive splenomegaly, which was frequently associated with thymic enlargement and/or lymph node swelling (Fig. 6B, upper panel and Supplementary Table 1). Leukemia was diagnosed as either acute myeloid leukemia (AML) or T-cell acute lymphoblastic leukemia (T-ALL) according to the results of flow cytometric and gene rearrangement analyses (Fig. 6B, lower panel, Supplementary Fig. 7, and Supplementary Table 1).

We then analyzed virus integration sites in tumor tissues from *Eed*^+/*Δ*^ + MOL4070A leukemic mice via inverse PCR (iPCR, Supplementary Table 2)^19^. We identified *Lmo2*, a T-cell-associated oncogene^20^, in several independent samples (No. 1, 3, and 5) and also detected leukemia-associated genes, such as *Fli1 (Friend leukemia virus-induced erythroleukemia-1*) and *Mecom (Mds1* and *Evi1* complex locus, also known as *Evi1*)^21^,^22^. Expression analysis of these genes in *Eed*^+/*Δ*^ + MOL4070A tumors revealed that although no obvious upregulation was observed for *Fli1*, enhanced expression of *Lmo2* and *MECOM (Evi1*) was detected (No. 8 and No. 6, respectively) (Fig. 6C). As coincident virus integration and gene upregulation were observed in the *Evi1* gene (Fig. 6C), we examined a possible synergistic effect of *Eed* haploinsufficiency with *Evi1* overexpression via virus-mediated gene transfer. c-kit^+^ hematopoietic cells from *control* or *Eed*^+/*Δ*^ mice were infected with an *Evi1-IRES-EGFP-*expressing retrovirus and were transplanted into irradiated syngeneic mice (Fig. 6D, upper panel). As shown in the lower panel of Fig. 6D, half of the recipients of *Evi1-*expressing *Eed*^+/*Δ*^ cells (*Eed*^+/*Δ*^ + *Evi1-IRES-EGFP*) developed acute leukemia, whereas none transplanted with *Evi1-*expressing control cells (*Ctrl* + *Evi1-IRES-EGFP*) exhibited hematopoietic abnormalities (*p* < 0.05). Macroscopically, diseased *Eed*^+/*Δ*^ + *Evi1-IRES-EGFP* mice exhibited massive splenomegaly, as observed in *Eed*^+/*Δ*^ + MOL4070A mice (Fig. 6E, left panel and Supplementary Table 3). Leukemic cells expressed myeloid antigens (Gr1 and Mac1) and were positive for EGFP, indicating that the leukemia met the diagnosis of acute leukemia and originated from virus-infected and transplanted *Eed*^+/*Δ*^ cells (Fig. 6E, right panels and Supplementary Table 3). These results indicated that *Evi1* overexpression in association with *Eed* haploinsufficiency promoted progression to acute leukemia.

To investigate the molecular mechanism underlying leukemic susceptibility by *Eed* haploinsufficiency, we examined the gene expression profiles of *control* and *Eed*^+/*Δ*^ LSK cells using RNA sequencing. Although we could not identify significantly enriched KEGG pathways using GSEA (FDR < 0.25 and Nominal p value < 0.05), we detected suppressed expression of several genes implicated in leukemogenesis, such as *Junb, Bcl11b, Tcf3 (E2A*), and *Sfpi1 (PU*.*1*) [fold changes in RNA expression level (RPKM; reads per kilobase of exon per million mapped sequence reads, *Eed*^+/*Δ*^ versus *Eed*^+/+^), 0.12, 0.25, 0.27 and 0.53, respectively].

## Discussion

EED is a non-catalytic but an essential subunit of PRC2 that regulates histone H3K27 methylation and contributes to transcriptional repression^23^,^24^. The enzymatic activity of PRC2 is principally mediated by the methyltransferase EZH2. However, previous attempts to clarify the biological role(s) of PRC2 by ablating *EZH2* function have achieved limited progress, particularly in adult hematopoiesis, likely due to the compensatory role of EZH1, an EZH2 homolog^25^. In this article, by generating *Eed*^*Δ*/*Δ*^ mice, we found that acquired EED deficiency almost completely abolished PRC2 function, leading to marked suppression of the global methylation status of H3K27 (Fig. 1A). Of note, protein expression levels of other PRC2 components, EZH2 and SUZ12, were markedly reduced by EED ablation (Fig. 1A), strongly suggesting that the assembly of EED, EZH2, and SUZ12 to form PRC2 is important in the protein stability of each PRC2 component, as suggested in previous studies^26^,^27^.

Acquired loss of EED and subsequent downregulation of H3K27 methylation levels resulted in premature lethality in *Eed*^*Δ*/*Δ*^ mice, which was associated with emaciation and a marked decrease in hematopoietic cells (Fig. 1B–E, left panel). BM analysis revealed marked reductions in LSK cells from *Eed*^*Δ*/*Δ*^ mice (Fig. 1E, middle and right panels), and a competitive repopulation assay revealed the severely impaired ability of *Eed*^*Δ*/*Δ*^ hematopoietic cells to reconstitute the hematopoietic cell compartment (Fig. 2). These results collectively indicate that EED-mediated maintenance of H3K27 methylation in the hematopoietic system is required for not only the proper differentiation but also the functional integrity of HSPCs.

A previously reported detailed study of EED in hematopoietic development used *Eed*^*flox*/*flox*^, *Vav*^*Cre*+^ (*Eed*^*KO*^) mice^28^. In this study, *Eed*^*KO*^ mice were born normally but exhibited a profound reduction in lineage-committed progenitors and perturbations in the differentiation and repopulation abilities of HSPCs, which closely resembled the phenotypes of our *Eed*^*Δ*/*Δ*^ mice (Figs 1 and 2). In that paper, the authors claimed that the decrease in hematopoietic cells resulted from increased cell death, based on enrichment of the pro-apoptotic genes by GSEA. However, our attempts to detect early primary changes in *Eed*^*Δ*/*Δ*^ HSPCs revealed that although the cell number of whole BM was significantly reduced, those in total and CD34^+^ LSK fractions were significantly increased and those in CMP and MEP fractions were significantly decreased (Supplementary Fig. 3). In accordance with this, the percentage of Annexin V^+^ cells was not altered in the LSK fraction, but significantly decreased in the Lin^−^ fraction and significantly increased in the Lin^+^ fraction in *Eed*^*Δ*/*Δ*^ mice compared with *control*s (Supplementary Fig. 8A). In addition, analyses of gene expression profiles including KEGG analysis in *Eed*^*Δ*/*Δ*^ LSK cells also did not detect any apparent upregulation of apoptosis-associated genes (Supplementary Fig. 8B). Moreover, the marked reduction in hematopoietic cells observed in *Eed*^*Δ*/*Δ*^ mice could not be rescued when we crossed *Eed*^*Δ*/*Δ*^ mice with *p53* KO mice to yield *Eed*^*Δ*/*Δ*^, *p53* KO compound mice, in which *p53*, an inducer of apoptotic genes, was absent (Supplementary Fig. 8C). These findings indicate that in *Eed*^*Δ*/*Δ*^ mice, primitive hematopoietic cells were retained or rather increased but lineage-committed cells were reduced, possibly due to increased apoptosis, and the latter would be responsible for the marked decrease in whole BM cells. The reason for the discrepancy between our results and the previous study^28^ remains unclear, but it might be attributable to the period when EED was deleted and when the analysis was performed. In the preceding study, *Eed* was deleted inherently, whereas in our study, *Eed* deletion was acquired and analyzed in the early stage. Alternatively, this discrepancy might have resulted from the cell types used in these studies. For gene expression analysis, the former study used LT-HSCs, whereas our study used LSK cells.

Notably, *Eed*^*Δ*/*Δ*^ HSPCs exhibited abnormal cell cycle patterns and demonstrated by BrdU incorporation and Pyronin-Y staining. Therefore, acquired EED deletion promoted cell cycle progression, as demonstrated by the increase in the S phase, but simultaneously inhibited cell cycle entry, as evidenced by the increase in the Pyronin Y-negative ratio in CD34^+^ cells (Fig. 3). In addition, GSEA revealed that the cell adhesion molecules gene set, which includes enhanced expression of cell attachment proteins, such as integrins, cadherins, selectins, and claudins, was most enriched in *Eed*^*Δ*/*Δ*^ LSK cells (Fig. 4A). In accordance with this, *in vitro* cell adhesion assay demonstrated that compared with *control* cells, *Eed*^*Δ*/*Δ*^ cells exhibited a significantly higher ability to adhere to fibronectin (Fig. 4B).

In BM, HSCs reside in a region with a special environment known as the BM niche. In this environmental niche, HSCs are surrounded by various types of cells including osteoblasts, mesenchymal cells, and vascular cells that coordinately maintain HSC quiescence through soluble factors and cell–cell interactions^29^. Thus, our finding that *Eed*^*Δ*/*Δ*^ HSPCs exhibited enhanced cell adhesion ability (Fig. 4) led us to the idea that EED deficiency promotes the adhesion of HSCs to environmental cells in the niche, thereby inhibiting the release of HSCs from the niche. Previous reports demonstrated that the molecular signature most significantly associated with HSC quiescence is the upregulation of cell adhesion genes^13^. These findings strongly postulate that although EED deficiency promotes cell cycle progression and accelerated cell proliferation, it also enhances HSC quiescence and inhibits cell cycle entry. These conflicting events eventually decrease the number of proliferating hematopoietic cells due to the limited supply of progenitor cells from the quiescent HSC pool, resulting in the depletion of lineage-committed and circulating hematopoietic cells. The impaired repopulation activity of *Eed*^*Δ*/*Δ*^ hematopoietic cells would also be attributed to this abnormal cell cycle. *Eed* deficiency enhances cell cycle progression in HSCs detached from the niche and consequently confers impaired repopulation ability upon these cells, as shown in previous reports that demonstrated a reverse effect of the cell cycle on the repopulating ability of HSCs^30^,^31^.

We also investigated the effect of *Eed* suppression on hematopoiesis using *Eed*^+/*Δ*^ mice and *siEED*-transduced human HSPCs. PB smears of *Eed*^+/*Δ*^ mice and *siEED*-transduced human CD34^+^ cord blood cells exhibited dysplasia in multilineage closely resembling the phenotype of MDS, indicating that suppressive expression EED underlies the morphological abnormalities observed in MDS and related neoplasm.

The competitive repopulation assay revealed a slight proliferative advantage of *Eed*^+/*Δ*^ LSK cells, as described in previous studies using *Eed*-haploinsufficient or *Eed*-hypomorphic hematopoietic cells^28^,^32^. In addition, by employing MOL4070A-induced insertional mutagenesis, we demonstrated that *Eed*^+/*Δ*^ hematopoietic cells exhibited susceptibility to leukemic transformation (Fig. 6A,B). Notably, whereas MOL4070A is capable of inducing not only myeloid but B-lymphoid and T-lymphoid leukemias^18^, the diseases that developed in MOL4070A-infected *Eed*^+/*Δ*^ mice were exclusively classified as either of the myeloid or T-lymphoid lineage (Supplementary Table 1). These phenotypes coincide with those observed in human hematopoietic diseases with *Eed* mutations^10^,^11^,^12^, strongly suggesting that our experimental system recapitulates the leukemogenic process induced by *Eed* haploinsufficiency.

We identified several leukemia-associated genes as retroviral insertion sites and demonstrated that overexpressed *Evi1* cooperated with *Eed* haploinsufficiency to develop AML (Fig. 5C–E). This finding strongly suggests that overexpressed EVI1 functions as a disease-promoting factor in myeloid disorders with PRC2 dysfunction. In fact, the search of the Cancer Genome Atlas (TCGA) database revealed that AML samples with high expression of Evi1 were significantly associated with low expression of EZH2, indicating a cooperative role of overexpression of *Evi1* and impaired function of PRC2 in the development of AML (manuscript in submission). In addition, a previous study that analyzed PRC2 mutations in myeloid malignancies reported that samples with inactivation and/or hypomorphic mutations in *EZH2* gene frequently carried concomitant mutations in the *Tet2, Asxl1*, and *Runx1* genes^17^. Therefore, it is strongly suggested that mutations and/or deregulated expression of epigenetic modifiers (ex. *Tet2* and *Asxl1*) and transcription factors (ex. *Runx1* and *Evi1*) may cooperate with dysfunction of PRC2 and promote progression of myeloid diseases. Regarding leukemias with a T-cell phenotype, PRC2 genes were reported to be frequently mutated in early T-cell precursor acute lymphoblastic leukemia (ETP ALL), a subtype of T-ALL with poor prognosis^33^. However, in contrast to ETP ALL that is characterized by the lack of T-lineage markers and aberrant expression of myeloid and HSC markers, our T-ALL samples expressed CD4 and/or CD8 markers (No. 2, 4, 5, and 7 in Supplementary Table 1). This suggests that T-cell leukemias developed in *Eed*^+/*Δ*^ + MOL4070A may represent not ETP ALL but rather adult T-ALL with PRC2 mutations, which accounts for approximately 25% of T-ALL samples^34^.

Concerning the underlying molecular mechanisms, although we could not identify significantly enriched KEGG gene sets using the RNA-seq results from *Eed*^+/*Δ*^ LSK cells, we detected altered expression of several genes implicated in leukemogenesis. For example, downregulation of *Junb (Eed*^+/*Δ*^/*control* = 0.12) is causative for myeloid leukemia, and suppressed expression of *Bcl11b* and *Tcf3 (E2A*) (*Eed*^+/*Δ*^/*control* = 0.25 and 0.28, respectively) is involved in the development of T-ALL (DRX046261 and DRX046262 in the DDBJ (DNA Data Bank of Japan) BioSample)^35^,^36^,^37^. Therefore, dysfunction of *Eed* might contribute to a myelodysplastic and leukemic predisposition, possibly through the altered expression of these downstream target genes.

**Figure 7:**
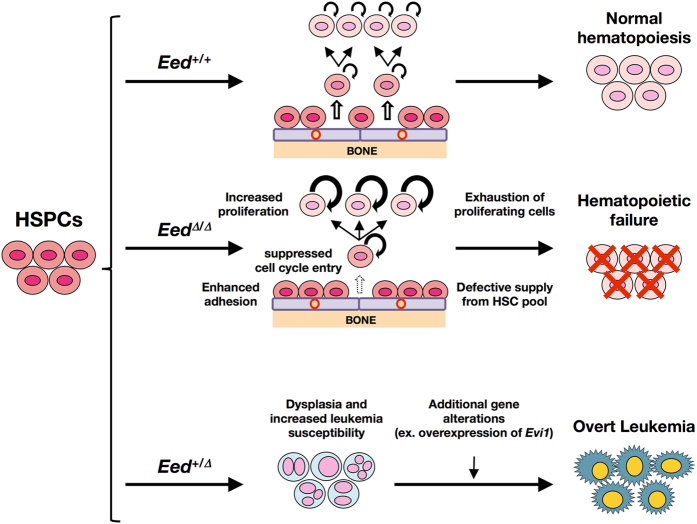
Schematic models of the biological implications of *Eed* deficiency and haploinsufficiency with respect to normal hematopoiesis and leukemogenesis. *Eed* deficiency results in hematopoietic failure, through exhaustion of proliferating hematopoietic cells and defective supply from HSC pool, possibly due to increased proliferation, suppressed cell cycle entry, and enhanced adhesion. In contrast, *Eed* haploinsufficiency induces dysplasia and increases susceptibility to leukemia with and progression to overt leukemia with additional gene alterations, such as *Evi1* overexpression.

In this article, we generated *Eed*-deficient mice and investigated the dose-dependent roles of EED in adult hematopoiesis and leukemogenesis (summarized in Fig. 7). Our findings, together with the results reported by another group^28^, demonstrate the complex and sophisticated roles of EED in both normal and abnormal hematopoiesis. Further research will be needed to clarify the roles of EED in various cell types within the hematopoietic hierarchy as well as different developmental stages and cell lineages.

## Methods

### Mice and human cells

*Cre*^*ERT2*+^ mice (C57BL/6-Gt(ROSA)26Sortm1(cre/Est1)Arte) were purchased from Taconic Biosciences (Hudson, NY, USA). Human CD34^+^ cord blood cells were purchased from RIKEN BRC (Tokyo, Japan). All mouse experiments were approved by the Hiroshima University Animal Research Committee (permission No. 22-175) and were carried out in strict accordance with the Guide for the Care and Use of Laboratory Animals of the Committee. The experiments using CD34^+^ cord blood cells were approved by the Ethical Committee of Hiroshima University (permission No. 988) and were performed in strict accordance with the guidelines of the Committee.

### Construction of conditional knockout vector and generation of *Eed* ^*flox*/*flox*^ mice

A bacterial artificial chromosome (BAC) clone containing the mouse *Eed* gene was purchased from the BACPAC Resource Center, Children’s Hospital Oakland Research Institute (Oakland, CA, USA). Fragments spanning the *EcoR*V site in intron 1 to the *Kpn*I site in intron 5 and from the *Apa*I site in intron 6 to the *EcoR*I site in intron 8 were used as the 5′ and 3′ arms of the targeting vector, respectively. A fragment spanning the *Kpn*I site in intron 5 to the *Apa*I site in intron 6 and containing exon 6 was floxed and inserted between the 2 arms, together with an *Frt*-flanked *Neomycin-resistance* gene. A *diphtheria toxin A (DTA*) gene was attached to the 5′ end of the vector as a negative selector. Embryonic stem (ES) cell electroporation and screening were performed using 1 × 10^5^ KY1.1 ES cells (kindly provided by Dr. Junji Takeda, Osaka University, Japan) and 30 μg of linearized vector, as previously described^38^. Individual candidate clones were screened via 5′ and 3′ genomic Southern blotting. Correctly targeted ES cells were microinjected into the blastocysts derived from C57BL/6 × BDF1 mice, and the resultant chimeric male mice were crossed with C57BL/6 female mice to transmit mutant allele to progeny. The *Neo resistance* gene was removed by crossing heterozygotes with CAG/FLPe transgenic mice (RIKEN BRC, RBRC1834) to produce *Neo*-deleted offspring. Mice that were backcrossed to C57BL/6N for at least seven generations were used for experiments. *Cre*^*ERT2*+^ mice (C57BL/6-Gt(ROSA)26Sortm1(cre/Est1)Arte) were purchased from Taconic Biosciences (Hudson, NY, USA). Cre activation was achieved by intraperitoneal administration of tamoxifen (Sigma, St. Louis, MO, USA; 1 mg per 20 g mouse body weight [BW]) for 5 consecutive days. Mouse experiments were performed in strict accordance with the Guide for the Care and Use of Laboratory Animals of the Hiroshima University Animal Research Committee (permission No. 22-175).

### Pathological and Western blot analyses

Pathological and Western blot analyses were performed as previously described^38^. Antibodies used for Western blot included anti-EED, anti-EZH2, and anti-monomethyl-Histone H3 (Lys27) (Merck Millipore, Billerica, MA, USA); anti-dimethyl-Histone H3 (Lys27), anti-trimethyl-Histone H3 (Lys27), and anti-histone H3 (Cell Signaling, Danvers, MA, USA); and anti-SUZ12 (SantaCruz Biotechnology, Santa Cruz, CA, USA).

### RNA extraction and quantitative real-time polymerase chain reaction (qPCR)

Total cellular RNA was extracted from leukemic tissues using a TRIZOL reagent (Invitrogen) according to the manufacturer’s protocol. Purified RNA was treated with DNase I and reverse-transcribed using SuperScript II reverse transcriptase (Invitrogen); cDNAs were subjected to quantitative real-time PCR using the SYBR Green PCR Master Mix. Primer sequences were as follows: Mouse *Mecom (Evi1*); 5′-CTTGCAACAAAACCTGGAGAGTG-3′ and 5′-CACCAACATGCTGAGATCGAATG-3′, Mouse *Lmo2*; 5′-ATGTCCTCGGCCATCGAAAG-3′ and 5′-CGGTCCCCTATGTTCTGCTG-3′, Mouse *Fli1*; 5′-TTGATTCAGCATACGGAGCGG-3′ and 5′-GGGCCGTTCTTCTCATCCAT-3′, Mouse *HPRT*; 5′-GCTGGTGAAAAGGACCTCTCG-3′ and 5′-CCACAGGACTAGAACACCTGC-3′, Human *EED*: 5′-GTGACGAGAACAGCAATCCAG-3′ and 5′-TATCAGGGCGTTCAGTGTTTG-3′, Human *HPRT*: 5′-CCTCATGGACTAATTATGGACAG-3′ and 5′-GCAGGTCAGCAAAGAATTTATAG-3′.

### Flow cytometric analysis

Cells were stained with FITC-, PE-, Biotin-, APC-, or PE-Cy7-conjugated anti-Ly5.1, anti-Ly5.2, anti-Gr1, anti-Mac1, anti-B220, anti-Thy1.2, anti-Ter119, anti-Sca1, anti-c-kit, anti-CD4, anti-CD8, anti-CD16/32, anti-CD34, anti-CD135, and anti-CD150 antibodies (BD Biosciences, San Jose, CA, USA or eBioscience, San Diego, CA, USA). Detection of HSPCs was performed on a BD FACSCanto™ II (BD Biosciences). The surface marker phenotypes of LSK, LT-HSC (long-term HSC), ST-HSC (short-term HSC), MPP (multipotent progenitor), CMP (common myeloid progenitors), GMP (granulocyte/macrophage progenitors), and MEP (megakaryocyte erythrocyte progenitor) are listed in Supplementary Table 4.

### Competitive repopulation assay

Intravenous injection of 2.5 × 10^6^ C57BL/6-CD45.2 (Ly5.2) BM mononucleated cells from 8-week-old *Eed*^*flox*/*flox*^, *Cre*^*ERT2*−^ and *Eed*^*flox*/*flox*^, *Cre*^*ERT2*+^ mice into 8.5-Gy-irradiated 8-week-old C57BL/6-CD45.1 (Ly5.1) recipient mice together with 5 × 10^5^ competitor cells from 8-week-old C57BL/6-CD45.1 (Ly5.1) mice was performed first. 4 weeks after BM transplantation, BM repopulation of the recipient mice were confirmed by calculating donor chimerism using flow cytometry. 2 days after the confirmation, tamoxifen was administrated intraperitoneally for 5 consecutive days. PB cells of the recipient mice were analyzed using flow cytometry and donor (Ly5.2) chimerism was calculated. In analysis of *Eed*^+/*Δ*^ mice, 2 × 10^3^ BM LSK cells from *control* and *Eed*^+/*Δ*^ mice with 5 × 10^5^ competitor cells were intravenously injected into recipients same condition as above.

### Cell cycle analysis

To determine cell proliferative activity, 3 doses of 5-bromodeoxyuridine (BrdU) (1mg/mice; Sigma) were injected intraperitoneally at 8-h intervals. 8-h after the last injection, LSK cells were collected and analyzed by flow cytometry as previously described^39^. To analyze G0 phase status, BM cells were incubated with 1 μg/ml Pyronin Y at 37 °C for 45 min. Using flow cytometer, LSK cells were analyzed as previously described^40^.

### Transcriptome analysis and data processing

mRNA-Seq libraries were prepared using the TruSeq RNA Sample Preparation Kit v2 (Illumina, San Diego, CA, USA). Transcriptome analysis was performed using a next-generation sequencer (GAIIx; Illumina) according to the manufacturer’s instructions. Generated sequence tags (>10,000,000 reads/sample) were mapped onto the mouse genomic sequence (UCSC Genome Browser, version mm10) using the sequence alignment program ELAND (Illumina). We compared the gene expression levels in LSK cells between 2 different genotypes (*control* vs. *Eed* homozygotes or *control* vs *Eed* heterozygotes) and identified genes that exhibited significant enrichments with GSEA.

### *In vitro* cell adhesion assay

A cell adhesion assay was performed as described previously^15^. Briefly, 2 × 10^3^ LSK cells from *control* and *Eed*^*Δ*/*Δ*^ mice at 2 days after tamoxifen administration were plated in a 96-well plate coated with RetroNectin (Takara Bio Inc., Otsu, Japan). After a 1-h incubation period, cells were gently washed twice, and residual attached cells were counted.

### MOL4070A infection and inverse PCR

Newborn mice were inoculated intraperitoneally with a MOL4070LTR (MOL4070A) retrovirus^18^ solution containing approximately 1 × 10^5^ virus particles; retroviral integration sites were identified using inverse PCR as previously described^19^.

### Retrovirus-mediated gene transfer

c-kit^+^ BM cells were infected with empty or *Evi1*-expressing *pMys-IRES-EGFP* retrovirus particles^41^. Here, 3 × 10^5^ cells were transplanted into 8.5-Gy-irradiated recipient mice, and hematopoietic parameters were continuously monitored.

### Transduction of *siRNA* in human CD34^+^ hematopoietic cells

Human CD34^+^ cord blood cells were cultured for 14 days in ReproHSC (ReproCELL Inc., Boston, MA, USA) containing recombinant human SCF serum (10 μg/ml; PeproTech, Rocky Hill, NJ, USA) in RetroNectin-coated dishes. 2 × 10^6^ CD34^+^ cells were subjected to 2 cycles of transduction of *Ctrl* and *EED si*RNAs (purchased from Dharmacon, Lafayette, CO, USA), as described^42^. 24 h later, cells were plated in MethoCult^TM^ (04434; StemCell Technologies, Vancouver, BC, Canada), and after 14 days of culture, erythroid burst-forming units (BFU-E), granulocyte-macrophage colony-forming units (CFU-GM), and granulocyte/erythrocyte/macrophage/megakaryocyte colony-forming units (CFU-GEMM) were scored and morphological changes of colony-forming cells were examined.

## Additional Information

**Accession codes:** RNA-seq data obtained in this study have been deposited under accession numbers DRX046251, DRX046252, DRX046261, and DRX046262 in the DDBJ (DNA Data Bank of Japan) BioSample.

**How to cite this article**: Ikeda, K. *et al*. Maintenance of the functional integrity of mouse hematopoiesis by EED and promotion of leukemogenesis by EED haploinsufficiency. *Sci. Rep.* **6**, 29454; doi: 10.1038/srep29454 (2016).

## Supplementary Material

Supplementary Information
