# Supplementary material for: Maintenance of the functional integrity of mouse hematopoiesis by EED and promotion of leukemogenesis by EED haploinsufficiency
Source: Sci Rep. 2016 Jul 19;6:29454. doi: 10.1038/srep29454 (PMC4949429; doi:10.1038/srep29454)
Supplement: Supplementary Information [file srep29454-s1.pdf]

Supplementary Information

Maintenance of the functional integrity of mouse hematopoiesis by EED and promotion of leukemogenesis by EED haploinsufficiency

Kenichiro Ikeda, Takeshi Ueda, Norimasa Yamasaki, Yuichiro Nakata, Yasuyuki Sera, Akiko Nagamachi, Takahiko Miyama, Hiroshi Kobayashi, Keiyo Takubo, Akinori Kanai, Hideaki Oda, Linda Wolff, Zen-ichiro Honda, Tatsuo Ichinohe, Akio Matsubara, Toshio Suda, Toshiya Inaba, and Hiroaki Honda

Supplementary Table 1. Characteristics of *Eed*$^{+/\Delta}$+MOL4070A leukemic mice

Mouse No. *	Age at disease (month)	PB parameters			Macroscopic † tumor sites	Surface markers	Gene rearrangements §		Diagnosis
		WBC ($\times 10^3$/ml)	Hb (g/dl)	Plt ($\times 10^4$/ml)			*IgH*	*TCRβ*	
1	7.0	81.0	10.7	14.1	Spl	Gr1$^+$, Mac1$^+$	G/G	G/G	AML
2	8.3	27.9	13.2	17.6	Spl, LN	Thy1.2$^+$ (CD4$^+$, CD8$^-$)	G/G	G/R	T-ALL
3[D]	8.9	5.0	8.5	50.7	Spl	N.D.	G/R	G/R	T-ALL s/o
4	9.6	20.8	11.9	30.2	Thy	Thy1.2$^+$, (CD4$^-$, CD8$^+$)	R/R	R/R	T-ALL
5	9.6	36.4	12.6	11.5	Thy, Spl	Thy1.2$^+$ (CD4$^+$, CD8$^+$)	G/G	R/R	T-ALL
6	9.6	25.4	11.4	21.8	Spl	Gr1$^+$, Mac1$^+$	G/G	G/G	AML
7	10.5	11.7	15.7	38.7	Thy, Spl	Thy1.2$^+$ (CD4$^+$, CD8$^+$)	G/R	G/R	T-ALL
8	10.5	7.6	11.0	51.0	Spl	Gr1$^+$, Mac1$^+$	G/R	G/G	AML

* D, found dead

† Thy, thymus; Spl, spleen; LN, lymph node

§ G, germline; R, rearran

Supplementary Table 2. Retrovirus integration sites identified by iPCR

Mouse No.	Mouse gene	Chromosomal No.	Integration site	Gene description
1	Dedd2	chr7(qA3)	5' up 20kb	death effector domain-containing DNA binding protein 2
	Pak2	chr16(qB2)	1st intron	p21 protein (Cdc42/Rac)-activated kinase 2
	Dedd2	chr7(qA3)	5' up 20kb	death effector domain-containing DNA binding protein 2
	Nup107	chr10(qD2)	26th intron	nucleoporin 107
	***Lmo2**	**chr2(qE2)**	**5' up 20kb**	**LIM domain only 2, transcript variant 1**
	Gng7	chr10(qC1)	2nd intron	guanine nucleotide binding protein (G protein), gamma 7, transcript variant 1
	Klf13	chr7(qA3)	3' down 20kb	death effector domain-containing DNA binding protein 2
	Lrp5	chr7(qC)	5' up 25kb	Kruppel-like factor 13
	Cacnb3	chr18(qE)	1st intron	low density lipoprotein receptor-related protein 5
	Atp6v0c	chr6(qB1)	5' up 100kb	ATPase, H+ transporting, lysosomal V0 subunit C
	Poli	chr18(qE2)	5' up 100kb	polymerase (DNA directed), iota, transcript variant 1
	Pald1	chr10(qB4)	1st intron	phosphatase domain containing, paladin 1
	Kazald1	chr19(qC3)	2nd intron	Kazal-type serine peptidase inhibitor domain 1
	Ly6c2	chr15(qD3)	5' up 3kb	lymphocyte antigen 6 complex, locus C2
2	C1galt1c1	chrX(qA3.3)	5' up 10kb	C1GALT1-specific chaperone 1 (C1galt1c1)
	Gfi1	chr5(qF)	5' up 7kb	growth factor independent 1 (Gfi1), transcript variant
	Pklr	chr3(qF1)	5' up 2kb	pyruvate kinase liver and red blood cell (Pklr), nuclear gene encoding mitochondrial protein, transcript variant 1
	Snx5	chr2(qG1)	3' dpwn 3kb	sorting nexin 5 (Snx5), transcript variant 2
	***Fli1**	**chr9(qA4)**	**1st intron**	**Friend leukemia integration 1(Fli1)**
	Psen2	chr1(qH4)	1st intron	presenilin 2 (Psen2), transcript variant 2
	B9d2	chr7(qA3)	1st intron	B9 protein domain 2 (B9d2)
	Psen2	chr1(qH4)	1st intron	presenilin 2 (Psen2), transcript variant 2
	Alcam	chr16(qB5)	5' up 10kb	activated leukocyte cell adhesion molecule (Alcam)
	Pigr	chr1(qE4)	7th intron	polymeric immunoglobulin receptor (Pigr)
	Srr	chr11(qB5)	1st intron	serine racemase (Srr), transcript variant 1
	Sgk1	chr19(qA3)	3rd intron	serum/glucocorticoid regulated kinase 1 (Sgk1), transcript variant 1
	Fzd9	chr5(qG2)	5' up 10kb	fizzled homolog 9 (Drosophila) (Fzd9)
	Ttll10	chr4(qE2)	5' up 10kb	tubulin tyrosine ligase-like family, member 10 (Ttll10)
	Dapp1	chr3(qG3)	3rd intron	dual adaptor for phosphotyrosine and 3-phosphoinositides 1 (Dapp1)
	Pigr	chr1(qE4)	8th intron	polymeric immunoglobulin receptor (Pigr)
	B9d2	chr7(qA3)	1st intron	B9 protein domain 2 (B9d2)
3	Snx29	chr16(qA1)	13th intron	sorting nexin 29 (Snx29)
	Ssh2	chr11(qB5)	2nd intron	slingshot homolog 2 (Drosophila)
	Cyhr1	chr15(qD3)	2nd intron	cysteine and histidine rich 1 (Cyhr1), transcript variant 1
	P2rx4	chr5(qF)	5' up 5kb	purinergic receptor P2X, ligand-gated ion channel 4
	Msh6	chr17(qE4)	5' up 500bp	mutS homolog 6 (E. coli) (Msh6)
	Pitpnm2	chr5(qF)	1st intron	phosphatidylinositol transfer protein, membrane-associated 2 (Pitpnm2)
	Ndst4	chr3(qG1)	5' up 100kb	N-deacetylase/N-sulfotransferase(heparin glucosaminyl) 4 (Ndst4)
	***Lmo2**	**chr2(qE2)**	**5' up 10kb**	**LIM domain only 2 (Lmo2), transcript variant 4**
	Tsc22d1	chr14(qD3)	1st intron	TSC22 domain family, member 1 (Tsc22d1), transcript variant 1
	Ubxn11	chr4(qD3)	8th intron	UBX domain protein 11 (Ubxn11), transcript variant 1
	Gm9054	chr3(qF2.1)	5' up 1kb	predicted gene 9054 (Gm9054), non-coding RNA
	Stat5a	chr11(qD)	5' up 1kb	signal transducer and activator of transcription 5A (Stat5a), transcript variant 1
4	Trp53i11	chr2(qE1)	1st intron	transformation related protein 53 inducible protein 11 (Trp53i11)
	Krcc	chr11(qB1.2)	5' up 500bp	lysine-rich coiled-coil 1 (Krcc1)
	Tgtp1	chr6(qC1)	5' up 1kb	T cell specific GTP 1 (Tgtp1)
	Gse1	chr8(qE1)	5' up 50kb	genetic suppressor element 1 (Gse1), transcript variant 1
	Exoc6	chr19(qC2)	5' up 50kb	exocyst complex component 6 (Exoc6)
	Gse1	chr8(qE1)	5' up 50kb	genetic suppressor element 1 (Gse1), transcript variant 1
	AK018976	chr19(qC2)	3' down 20kb	adult male testis cDNA, RIKEN
	Tmen173	chr18(qB2)	5' up 10kb	transmembrane protein 173 (Tmen173)
	Ankrd46	chr15(qB3.1)	3' down 25kb	ankyrin repeat domain 46 (Ankrd46)
	Tom1l2	chr11(qB2)	9th intron	target of myb 1-like 2 (chicken) (Tom1l2), transcript variant 2
	Selplg	chr5(qF)	5' up 2kb	selectin, platelet (p-selectin) ligand (Selplg)
	Ankrd6	chr4(qA5)	2nd intron	ankyrin repeat domain 6 (Ankrd6), transcript variant 3
	Exoc6	chr19(qC2)	5' up 50kb	exocyst complex component 6 (Exoc6)
	Kiss1r	chr10(qC1)	5th exon	KISS 1 receptor (KISS1r)
	Shf	chr2(qE5)	2nd intron	Src homology 2 domain containing F (Shf)
	Pcyt1a	chr16(qB3)	1st exon	phosphate cytidylyltransferase 1, choline, alpha isoform (Pcyt1a), transcript variant 3
5	B3gntl1	chr11(qE2)	5' up 15kb	UDP-GlcNAc:betaGal beta-1, 3-N-acetylglucosaminyltransferase-like 1 (B3gntl1)
	Itgb2	chr19(qC1)	5' up 14kb	integrin beta 2 (Itgb2)
	***Lmo2**	**chr2(qE2)**	**5' up 20kb**	**LIM domain only 2 (Lmo2), transcript variant 4**
	Sema7a	chr9(qB)	1st intron	sema domain, immunoglobulin domain (Ig), and GPI membrane anchor, (semaphorin) 7A
	Sdk1	chr5(qG2)	20th intron	sidekick homolog 1 (chicken) (Sdk1)
	Parvb	chr15(qE2)	13th intron	parvin, beta (Parvb)
	Rab37	chr11(qE2)	2nd intron	RAB37, member of RAS oncogene family (Rab37), transcript variant 1
	Lrrc25	chr8(qB3.3)	2nd exon	leucine rich repeat containing 25 (Lrrc25)
	Metrn1	chr15(qE2)	5' up 13kb	meteorin, glial cell differentiation regulator-like (Metrnl)
	B3gntl1	chr11(qE2)	5' up 18kb	UDP-GlcNAc:betaGal beta-1, 3-N-acetylglucosaminyltransferase-like 1 (B3gntl1)
	Gabpb2	chr3(qF2.1)	1st exon	GA repeat binding protein, beta 2 (Gabpb2), transcript variant 1
	Spry1	chr3(qB)	3' down 10kb	sprouty homolog 1 (Drosophila) (Spry1)
6	Zfp36	chr7(qB1)	5' up 1.5kb	Zinc finger protein 36
	Klhl35	chr7(qE1)	2nd intron	kelch-like 35 (Dorosophila) (Klhl35)
	Fam71e1	chr7(qB3)	2nd intron	family with sequence similarity 71, member E1 (Fam71e1)
	Il2ra	chr2(qA1)	5' up 5kb	interleukin 2 receptor, alpha chain (Il2ra)
	***Mecom**	**chr3(qA3)**	**2nd intron**	**MDS1 and EVI1 complex locus (Mecom), transcript variant 1**
	Acot7	chr4(qE2)	1st intron	acyl-CoA thioesterase 7 (Acot7), transcript variant 1
	Map3k8	chr18(qA1)	3' down 25kb	mitogen-activated protein kinase kinase kinase 8 (Map3k8)
	Tmen108	chr9(qF1)	2nd intron	transmembrane protein 108 (Tmen108)
7	Phtf2	chr5(qA3)	1st intron	putative homeodomain transcription factor 2 (Phtf2)
	Asgr	chr11(qB3)	2nd intron	asialoglycoprotein receptor 2 (Asgr2)
	Oaz1	chr10(qC1)	5' up 1.5kb	ornitine decarboxylase antizyme 1 (Oaz1)
	Ywhaz	chr15(qB3)	3' down 40kb	tyrosine 3-monooxygenase-/tryptophan 5-monooxygenase activation protein, zeta polypeptice (Ywhaz), transcript variant 1
	Lrrc58	chr16(qB3)	5' up 800bp	leucine rich repeat containing 58 (Lrrc58)
	Csnk1e	chr15(qE1)	2nd intron	casein kinase 1, epsilon (Csnk1e)
	Ncam1	chr9(qA5.3)	5' up 100kb	neural cell adhesion molecule 1 (Ncam1), transcript variant 3
	Myl10	chr5(qG2)	5' up 500bp	myosin, light chain 10, regulatory (Myl10), transcript variant
	Slc11a1	chr1(qC3)	5th exon	solute carrier family 11 (proton-coupled divalent metal ion transporters), member 1 (Slc11a1)
	Lipc	chr9(qD)	1st intron	lipase, hepatic (Lipc)
	Dnmt3b	chr2(qH1)	5' up 10kb	DNA methyltransferase 3B, transcript variant 1
8	Arhgef28	chr11(qC)	3rd exon	chemokine (C-C motif) ligand 3
	Mrgpra3	chr9(qD)	1st intron	MAS-related GPR, member A3
	Mapkap1	chr7(qB4)	5' up 170kb	mitogen-activated protein kinase associated protein 1, transcript variant 1
	Wasf3	chr10(qA3)	3rd intron	WAS protein family, member 3
	Slc35b1	chr10(qA3)	2nd intron	solute carrier family 35, member 1
	Fam117b	chr1(qC2)	1st intron	family with sequence similarity 117, member B
	Bai1	chr1(qC2)	1st intron	brain-specific angiogenesis inhibitor 1
	Mroh4	chr15(qD3)	3' down 10kb	maestro heat-like repeat family member 4
	Rbm43	chr2(qC1.1)	3' down 10kb	RNA binding motif protein 43, transcript variant 1
	Irf1	chr11(qB1.3)	5' up 300bp	interferon regulatory factor 1, transcript variant 1
	Dapp1	chr3(qG3)	5' up 8kb	dual adaptor for phosphotyrosine and 3-phosphoinositides 1
	Csf1r	chr18(qE1)	5' up 8kb	colony stimulating factor 1 receptor

***leukemia-associated genes subjected to qPCR**

Supplementary Table 3. Characteristics of leukemias in *Eed*$^{+/\Delta}$+*Evi1-IRES-EGFP* mice

Mouse No.	Age after BMT (days)	PB parameters			Macroscopic tumor sites *	Surface markers	Diagnosis
		WBC ($\times 10^3/\mu l$)	Hb (g/dl)	Plt ($\times 10^4/\mu l$)			
1	23	1.9	5.4	46.8	Spl	$Gr1^+$, $Mac1^+$	AML
2	43	42.5	8.0	13.4	Spl	$Gr1^+$, $Mac1^+$	AML
3	106	36.6	13.7	19.4	Spl	$Gr1^+$, $Mac1^+$	AML
4	106	49.4	12.4	10.7	Spl	$Gr1^+$, $Mac1^+$	AML

* Spl, spleen

Supplementary Table 4. Surface markers to separate HSPC fractions

Fraction	Surface markers
LSK	c-kit^{+}, Sca-1^{+}, Lin^{-}
LT-HSC	c-kit^{+}, Sca-1^{+}, Lin^{-}, CD34^{-}, CD135^{-}
ST-HSC	c-kit^{+}, Sca-1^{+}, Lin^{-}, CD34^{+}, CD135^{-}
MPP	c-kit^{+}, Sca-1^{+}, Lin^{-}, CD34^{+}, CD135^{+}
CMP	c-kit^{+}, Sca-1^{+}, Lin^{-}, CD34^{+}, CD16/32^{low}
GMP	c-kit^{+}, Sca-1^{+}, Lin^{-}, CD34^{+}, CD16/32^{high}
MEP	c-kit^{+}, Sca-1^{+}, Lin^{-}, CD34^{-}, CD16/32^{-}

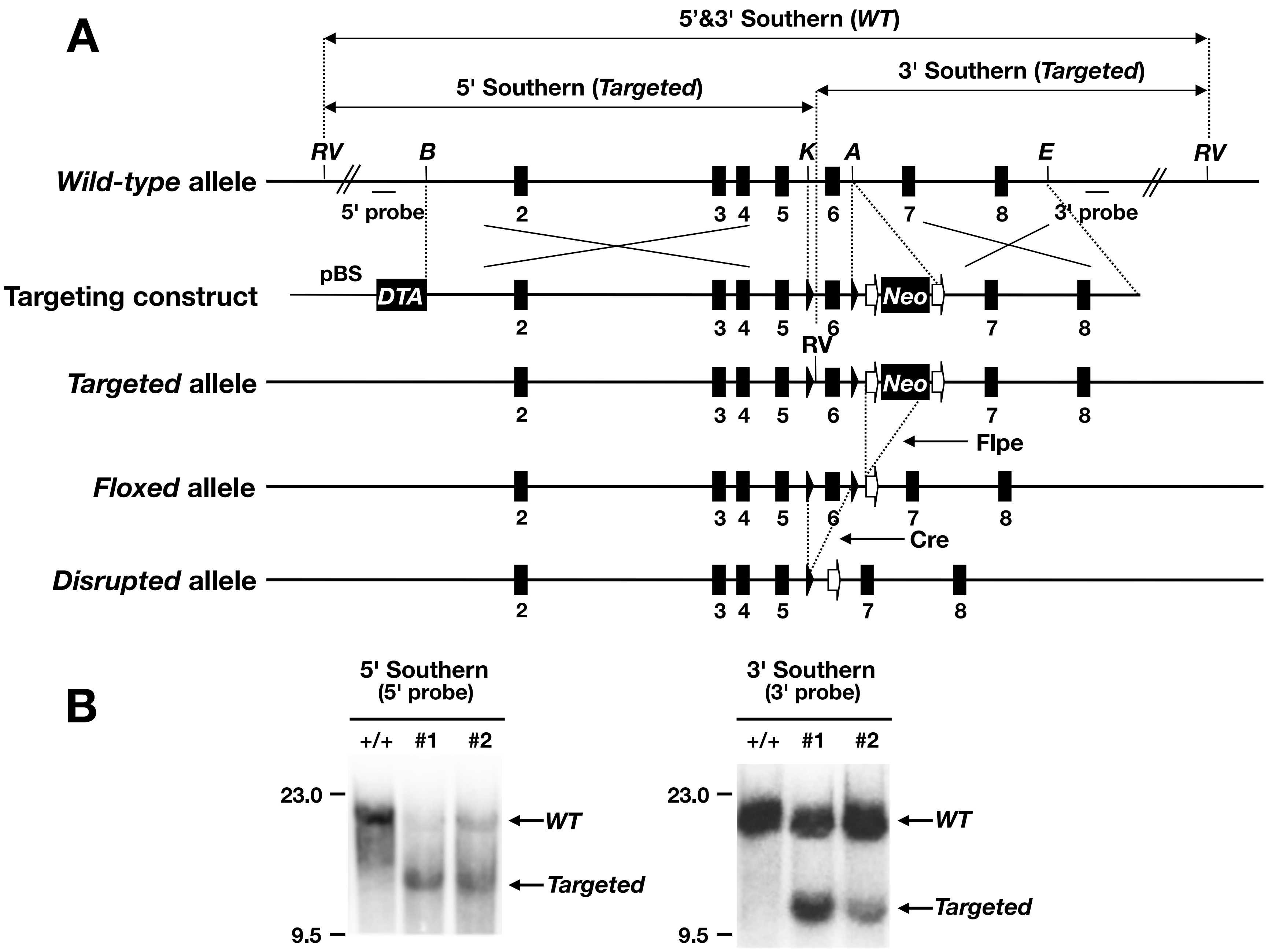

Supplementary Figure 1. Ikeda K. *et al.*

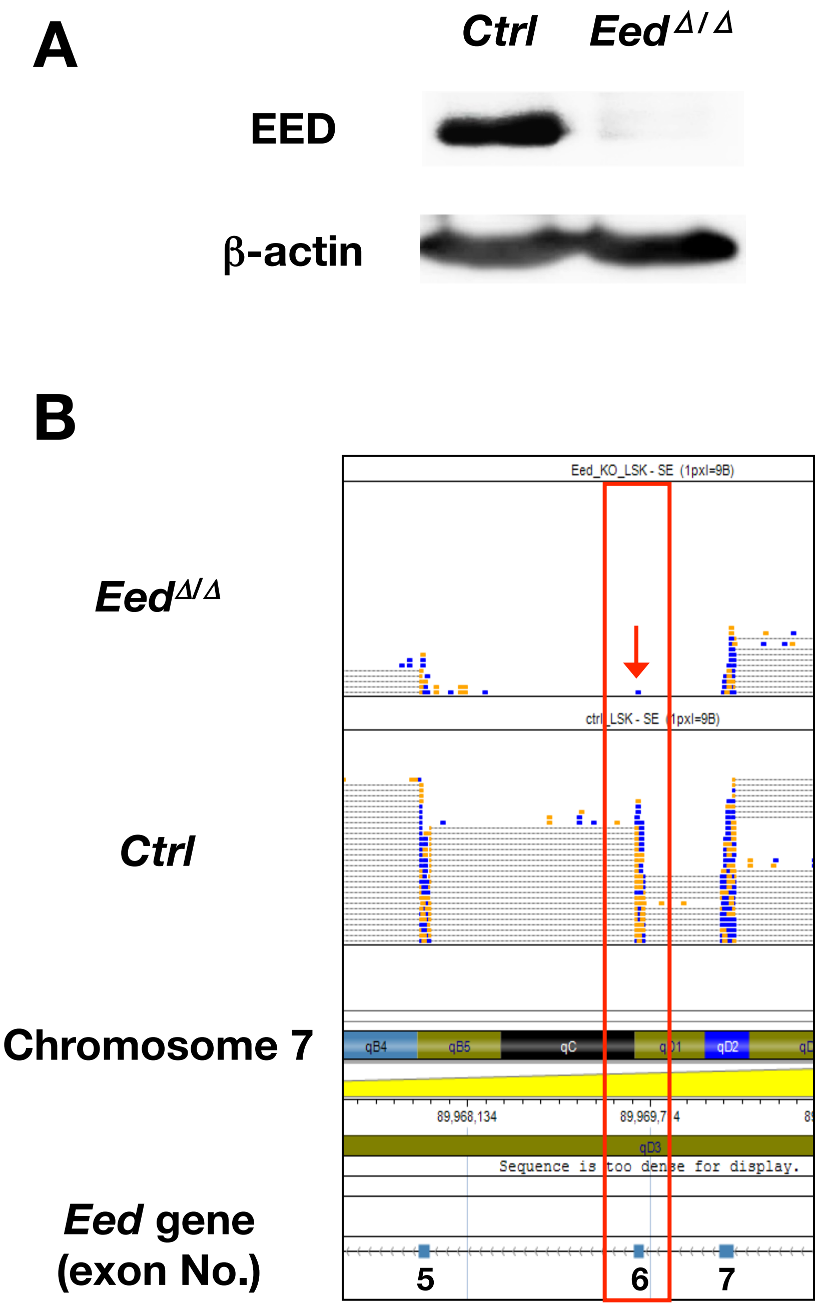

Supplementary Figure 2. Ikeda K. *et al.*

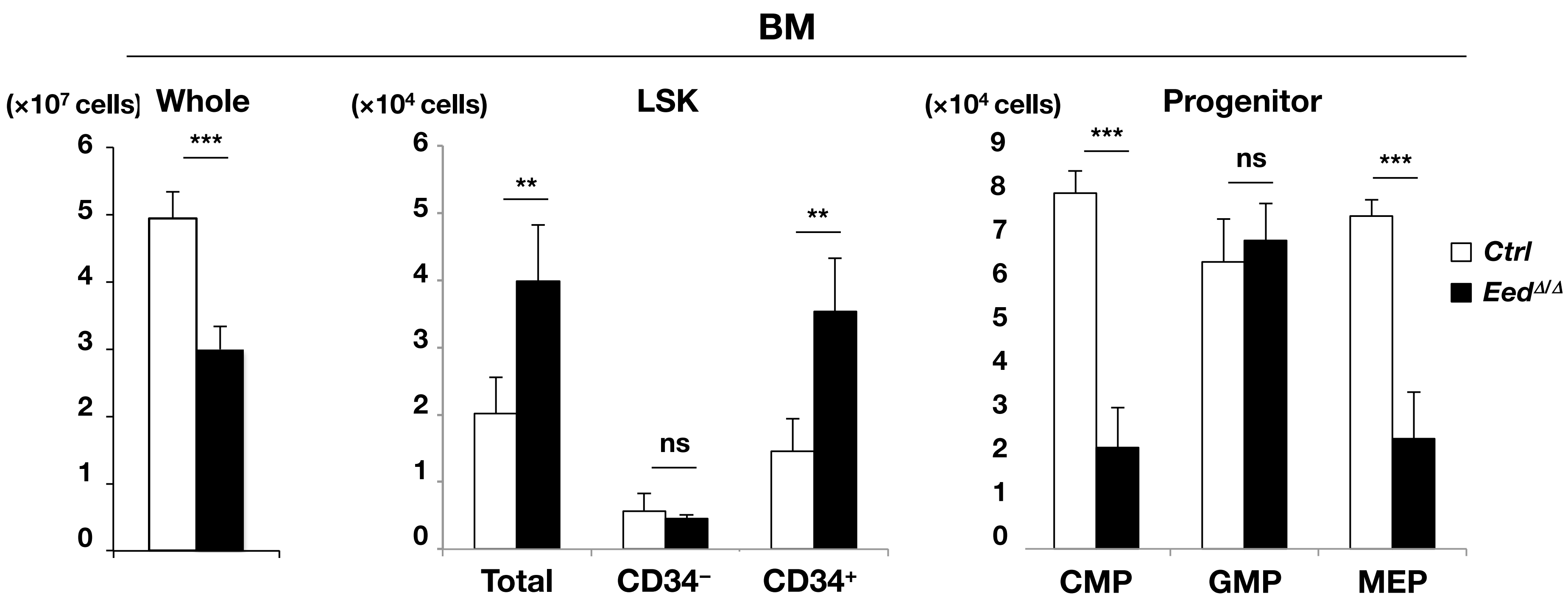

Supplementary Figure 3. Ikeda K. *et al.*

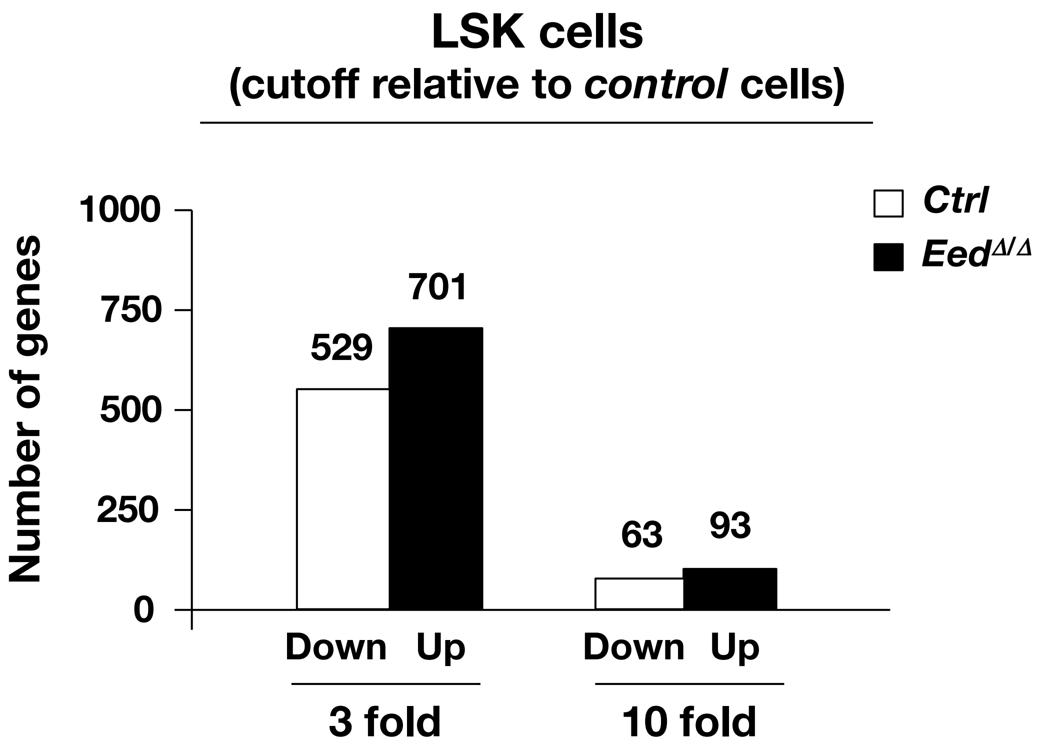

Supplementary Figure 4. Ikeda K. *et al.*

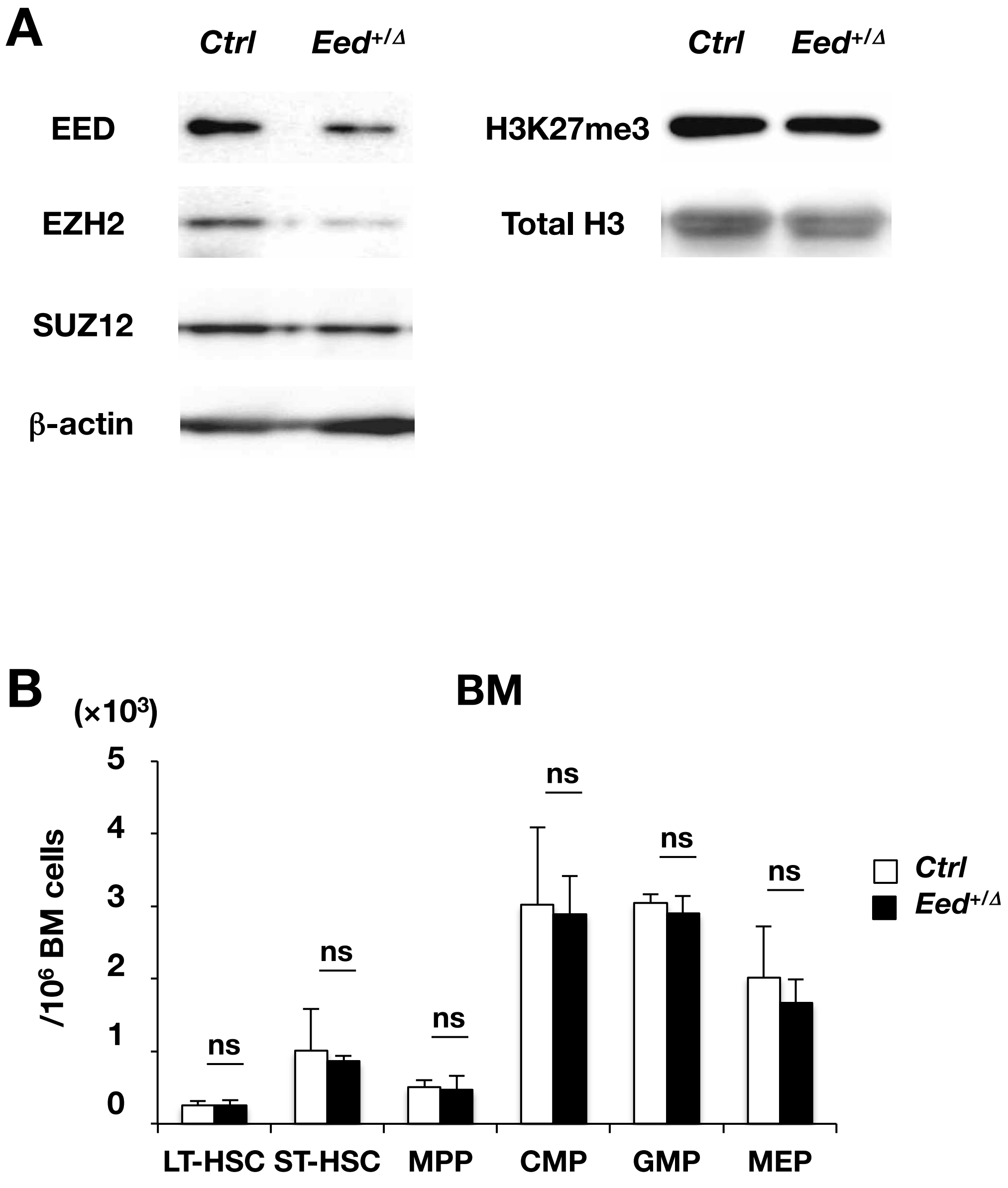

Supplementary Figure 5. Ikeda K. *et al.*

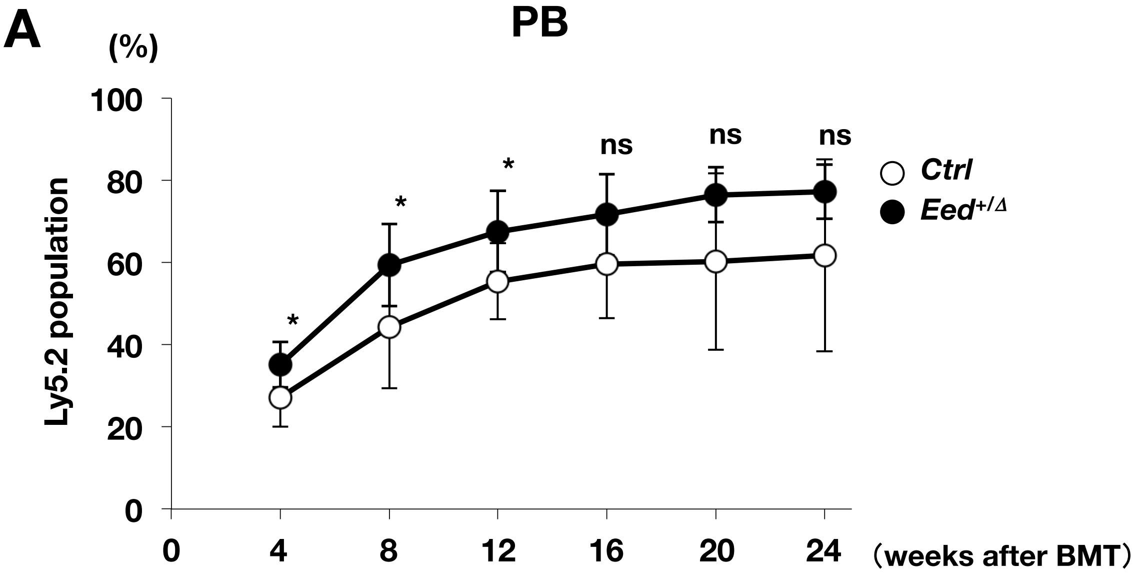

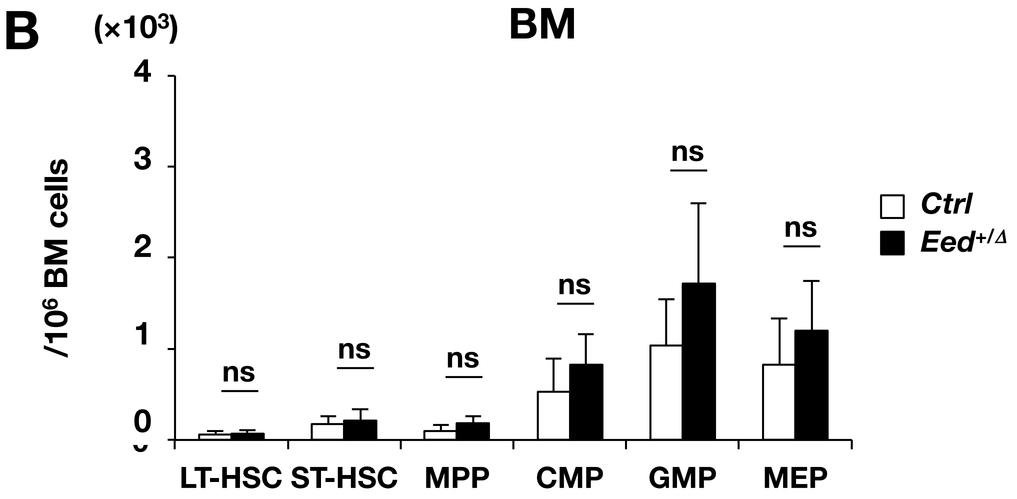

Supplementary Figure 6. Ikeda K. *et al.*

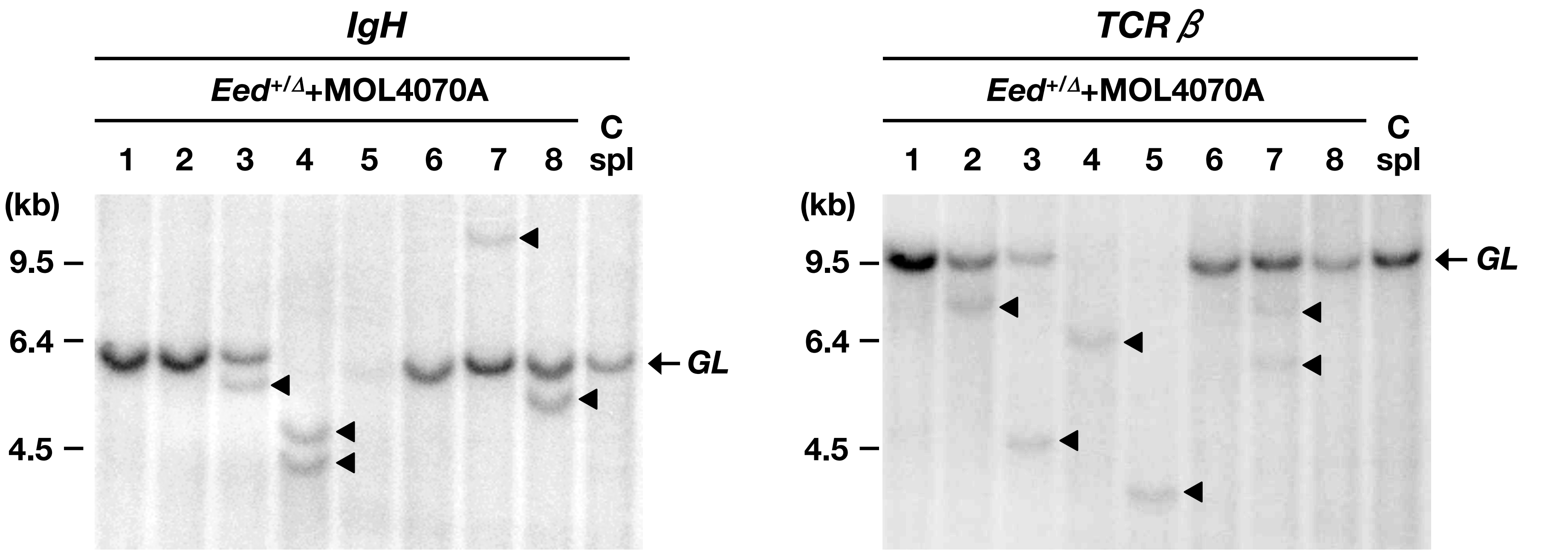

Supplementary Figure 7. Ikeda K. *et al.*

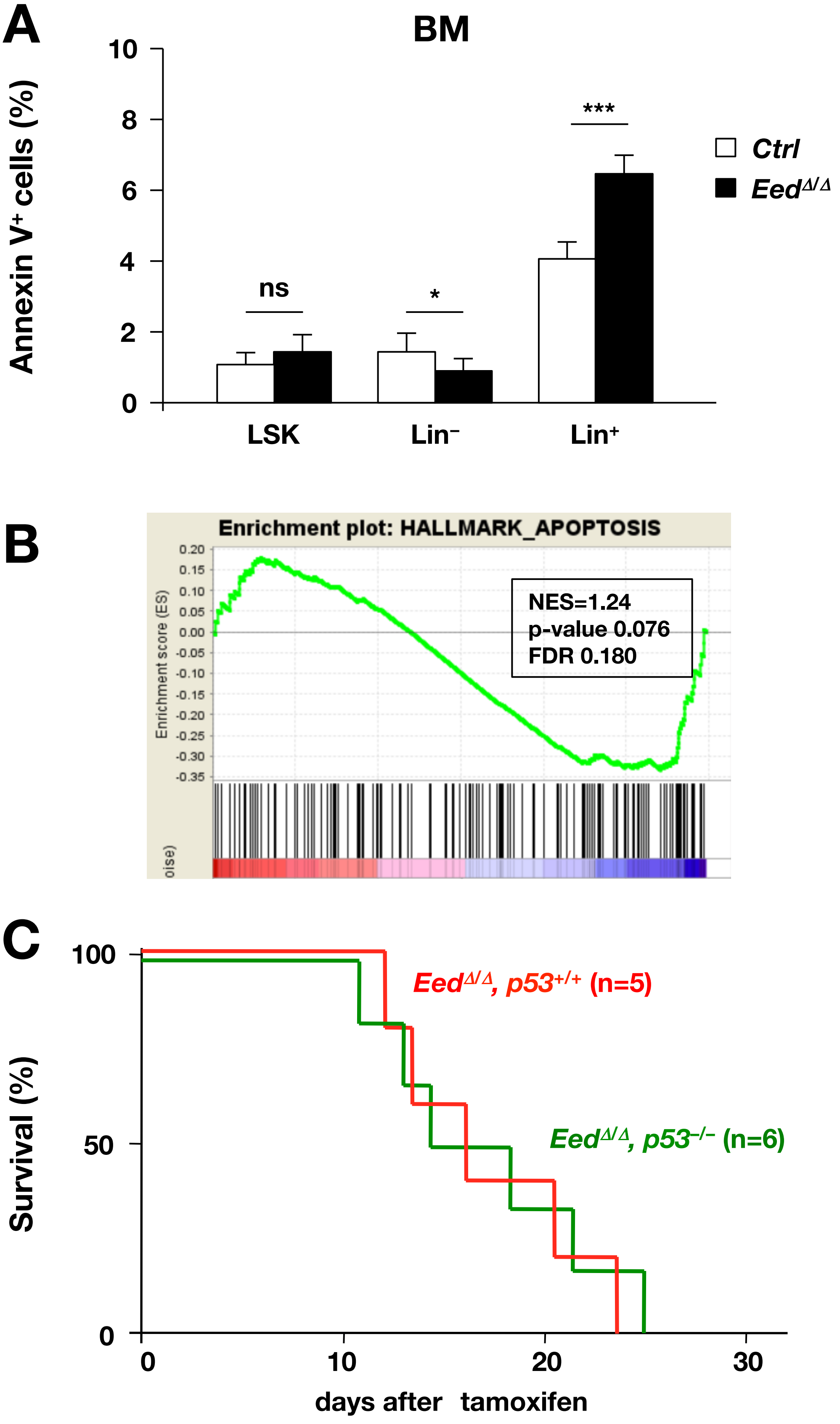

Supplementary Figure 8. Ikeda K. *et al.*

Legends of Supplementary Figures

Supplementary Figure 1. Targeting strategy and genotyping of ES clones. (A) Exon 6 of mouse *Eed* was encompassed by two *loxP* sites (black triangles), and a *Neo-resistance* gene (*Neo*) was flanked by two *Frt* sites (white arrows). After removing *Neo* using Flpe, exon 6 was deleted by Cre. The positions of genomic 5′ and 3′ probes during Southern blot analysis are shown. The restriction sites are *RV*, *EcoR*V; *B*, *BamH*I; *K*, *Kpn*I; *A*, *Apa*I; and *E, EcoR*I. (B) Homologously recombined embryonic stem cell clones (#1 and #2) identified via Southern blotting. *Wild-type* (*WT*) and *targeted* bands are indicated by arrows.

Supplementary Figure 2. Absence of EED protein and *Eed* exon 6-derived transcript in *Eed*$^{\Delta/\Delta}$ hematopoietic cells. (A) Western blot of EED in the BM cells of *control* and *Eed*$^{\Delta/\Delta}$ mice. (B) Accumulation of transcripts derived from *Eed* exons of *Eed*$^{\Delta/\Delta}$ and *control* LSK cells. *Eed* exon 6-derived transcripts are boxed and those of *Eed*$^{\Delta/\Delta}$ LSK cells are indicated by an arrow.

Supplementary Figure 3. Analysis of *Eed*$^{\Delta/\Delta}$ HSPCs at 2 days after tamoxifen administration. (A) Cell numbers of whole and LSK (total, CD34$^-$, and CD34$^+$) and progenitor (CMP, GMP, and MEP) fractions in the BM of *control* and *Eed*$^{\Delta/\Delta}$ mice at 2 days after tamoxifen administration. ns; not significant, **; $p < 0.01$, ***; $p < 0.001$.

Supplementary Figure 4. Analysis of RNA expression data of *Eed*$^{\Delta/\Delta}$ LSK cells. Numbers of genes downregulated and upregulated in *Eed*$^{\Delta/\Delta}$ LSK cells compared with control cells. Results of 3-fold and 10-fold cutoffs are shown.

Supplementary Figure 5. Hematopoietic analysis of *Eed*$^{+/\Delta}$ mice. (A)Western blot of EED and other PRC2 components, EZH2, and SUZ12 (left panels), and of

H3K27me3−me1 (right panels) in the spleens of *control* and *Eed*$^{+/\Delta}$ mice. (B) Analysis of HSPCs in the BM of *control* and *Eed*$^{+/\Delta}$ mice. ns; not significant.

Supplementary Figure 6. BMT analysis of *Eed*$^{+/\Delta}$ HSPCs. (A) Percentages of donor (*Ctrl* or *Eed*$^{+/\Delta}$)-derived cells in the peripheral blood of recipient mice. ns; not significant, *; $p < 0.05$. (B) Number of donor (*Ctrl* or *Eed*$^{+/\Delta}$)-derived HSPCs in the BM of recipient mice. ns; not significant.

Supplementary Figure 7. Analysis of gene rearrangements in tumors developed in *Eed*$^{+/\Delta}$ + MOL4070A mice. DNAs extracted from tumor tissues from *Eed*$^{+/\Delta}$ + MOL4070A mice and a control spleen (C Spl) were digested with *EcoR*I and blotted with an *IgH* (left panel) or a *TCRβ* probe (right panel). *Germline* (*GL*) and *rearranged* bands are indicated by arrows and arrowheads, respectively.

Supplementary Figure 8. Analysis of apoptosis of *Eed*$^{\Delta/\Delta}$ HSPCs. (A) Percentages in Annexin V^{+} cells in LSK, Lin^{-}, and Lin^{+} fractions. ns; not significant, *; $p < 0.05$, ***; $p < 0.001$. (B) GSEA analysis for apoptosis related genes. No significant enrichment was detected between *control* and *Eed*$^{\Delta/\Delta}$ LSKs. (C) Survival curves of *Eed*$^{\Delta/\Delta}$, *p53*$^{+/+}$ and *Eed*$^{\Delta/\Delta}$, *p53*$^{-/-}$ mice. ns; not significant.
